# An offer you cannot refuse: down-regulation of immunity in response to a pathogen's retaliation threat

**DOI:** 10.1111/jeb.12209

**Published:** 2013-08-09

**Authors:** O Restif

**Affiliations:** Disease Dynamics Unit, Department of Veterinary Medicine, University of CambridgeCambridge, UK

**Keywords:** adaptive dynamics, host–parasite interaction, theory

## Abstract

According to the Red Queen hypothesis, hosts and pathogens are engaged in an escalating coevolutionary arms race between resistance and virulence. However, the vast majority of symbionts colonize their hosts' mucosal compartments without triggering any immune response, resulting in durable commensal associations. Here, I propose a simple extension of previous mathematical models for antagonistic coevolution in which the host can mount a delayed immune response; in response, the symbiont can change its virulence following this activation. Even though the levels of virulence in both phases are assumed to be genetically determined, this simple form of plasticity can select for commensal associations. In particular, coevolution can result in hosts that do not activate their immune response, thus preventing phenotypically plastic pathogens from switching to a higher virulence level. I argue that, from the host's point of view, this state is analogous to the mafia behaviour previously described in avian brood parasites. More importantly, this study provides a new hypothesis for the maintenance of a commensal relationship through antagonistic coevolution.

## Introduction

Virtually all eukaryotic organisms are hosts to microorganisms in associations that combine various degrees of positive and negative effects on all the actors involved (Dethlefsen *et al*., 2007). Although antagonistic coevolution between pathogens and their hosts has been investigated for several decades (Frank, 1992), the attention of microbiologists has recently been drawn to the microbiota, the myriad symbionts that persistently colonize various parts of larger multicellular organisms. A major evolutionary puzzle is how hosts can simultaneously tolerate so many different species of symbionts and maintain the ability to detect and attack the pathogens that occasionally appear in their midst. Current research suggests that, although symbionts have evolved strategies to fend off immune defences, the composition of the microbiota is largely determined by the host's genotype (Fraune & Bosch, 2007; Royet *et al*., 2011).

There is growing evidence that certain symbionts provide their hosts with benefits, ranging from processing of nutrients (Akman *et al*., 2009) to defence against pathogens (Scarborough *et al*., 2005). Evolutionary theory suggests ways in which such mutualistic symbioses can be maintained, for example if hosts evolve strategies to punish less beneficial symbionts (West *et al*., 2002). However, it is unlikely that the thousands of bacterial species commonly found in the human gut (Qin *et al*., 2010) all confer benefits; many of them are probably commensals inhabiting a warm, resource-rich environment at little cost to the host (Hooper & Gordon, 2001). This raises the question of the evolution of commensalism, in the grey area between mutualism and parasitism.

Two main hypotheses have been proposed and explored theoretically to explain how parasitic symbionts can evolve towards commensalism. First, an important factor is the route of transmission of the symbiont: vertical transmission from parent to offspring aligns the interests of the host and the symbiont and is therefore expected to promote avirulence (Bull *et al*., 1991; Ferdy & Godelle, 2005); it is indeed a route used by various mutualistic symbionts (Leigh, 2010). Another hypothesis involves host tolerance, a phenomenon by which certain host genotypes can minimize the cost of pathogens to their own fitness (Roy & Kirchner, 2000). This has been described in plant (Kover & Schaal, 2002) and animal hosts (Råberg *et al*., 2007). However, coevolutionary models have shown that tolerance can actually select for more virulent pathogens (Restif & Koella, 2003; Best *et al*., 2010). In an interesting twist, tolerant hosts can actually harbour (at some cost) pathogens that will decimate less tolerant competitors, effectively using their symbionts as biological weapons (Brown *et al*., 2006).

Although the studies cited above have only considered fixed phenotypes, most infections, whether acute or persistent, actually go through several stages, often involving phenotypic plasticity of both the host and the symbiont. In animals (vertebrates or invertebrates), selective activation of the vast immune arsenal upon detection of bacterial molecules is subjected to a complex regulatory system, which effectively prevents unnecessary attack of resident symbionts (Royet *et al*., 2011). On the other side, various pathogens and parasites causing chronic infection can respond to stress within the host by adjusting their life-history traits (Reece *et al*., 2009), which can result in variations in virulence: among others, *Plasmodium vivax*, varicella zoster virus and *Mycobacterium tuberculosis* can alternate between acute and dormant phases.

In this study, I investigate some evolutionary consequences of phenotypic plasticity during the course of an infection. In particular, I ask whether a symbiont can coerce its host into down-regulating the activation of immune defences in exchange for a reduced virulence – thus reversing the classic idea of host-driven enforcement of cooperation (Leigh, 2010). This would represent an alternative explanation for the widespread occurrence of mild, commensal associations as described above. Most models developed to date have assumed fixed interactions between hosts and symbionts: the levels of immune defence and pathogenic damage as well as the infectivity to other hosts are assumed to remain constant for the duration of infection. Although Taylor *et al*. (2006) modelled the coevolution of plastic traits in a host and its symbiont, their negotiation framework implicitly assumes instantaneous changes in phenotypes at the beginning of the association. In contrast, Osnas & Dobson (2010) used a model with two stages of infection to study how the relative timings of disease and transmission affect virulence evolution, but did not consider host evolution.

Here, I develop a model where the host can mount a delayed immune response against a horizontally transmitted symbiont after the start of infection, and the symbiont can respond by varying the damage it causes to the host under the classic transmission–virulence trade-off. To use an anthropocentric analogy, I ask whether a symbiont can be selected to use mafia-style coercion to ensure its host's benevolence with the threat of lethal retribution for its disloyalty. This analogy has been used by evolutionary biologists to describe the behaviour of cuckoos and other brood parasites (Robert *et al*., 1999; Hoover & Robinson, 2007), and it has been suggested that it could apply to other parasitic systems (Soler *et al*., 1998). Yet this is, to my knowledge, the first attempt at formalizing the evolution of a mafia behaviour in a symbiotic association.

## Model description and analysis

### Population and infection dynamics

I use a simple extension of previous host–pathogen coevolutionary models. The host population follows a logistic growth model in the absence of pathogens: the effective birth rate per capita is equal to 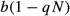, where *N* is the population size and *b* and *q* are positive parameters; the death rate is *m*; hence, the carrying capacity is given by *K* = (*b*−*m*)/(*bq*) (see Table S1 in the Appendix S1 for a complete list of symbols).

Upon infection, hosts go through two successive phases characterized by different recovery rates: 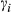 during the initial phase and 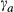 following activation of the immune response. By default, I assume that infection cannot be cleared while the immune response is inactive (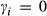). However, in the appendix, I show that the results presented here remain similar when 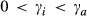. Activation of the immune response occurs at a rate *μ*. During the two successive phases, infected hosts suffer additional death rates 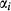 and 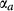, which depend on the genotype of the pathogen. Finally, transmission of the pathogen occurs by direct contact between hosts: the number of new infections per time unit follows mass action and is given by 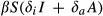, where *S*, *I* and *A* are the numbers of susceptible hosts, infective hosts in the initial phase and infected hosts in the active phase, respectively; 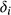 and 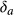 measure the respective infectiousness of the two phases; and *β* combines the hosts' susceptibility to infection and contact rate.

**Figure 1:**
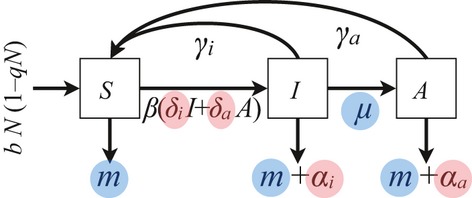
Schematic of the population dynamic models. Host evolution affects parameters highlighted in blue (*m* and *μ*) and pathogen evolution those in pink (*α* and *δ*).

Thus, the dynamics of the model (Fig. 1) with single genotypes of hosts and pathogens are described by the following set of ordinary differential equations (where *N* = *S* + *I* + *A*):

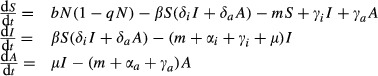
1

The pathogen's basic reproductive ratio is given by (see Appendix S2 for the derivation):

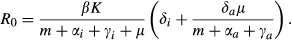


This expression is equivalent to that derived by Osnas and Dobson (2010). Conditions for the persistence of the pathogen can be obtained by solving the inequality 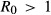.

### Pathogen evolution

For simplicity, I follow the well-studied assumption of a trade-off between virulence (as measured by disease-induced host mortality) and infectivity. Although not universal, this assumption has received theoretical and empirical support (see Alizon *et al*., 2009, for a detailed review). Its main advantage here is to allow comparison with a large set of existing theoretical models. More specifically, I consider two options, corresponding to pathogen evolution at either one locus or two.

In the single-locus model, the pathogen's traits are identical during both phases of infection: 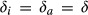 and 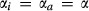. Virulence evolution is modelled by assuming that infectivity *δ* increases at a decelerating rate with virulence *α* across the range of possible pathogen genotypes. Using the relation 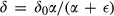, where 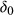 and *ε* are positive parameters, leads to the classical prediction that the pathogen reaches an evolutionarily stable (ES) level of virulence (Bremermann & Pickering, 1983; van Baalen & Sabelis, 1995).

In the two-locus model, also referred to below as the plastic virulence model, natural selection will optimize the two levels of virulence 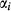 and 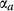 independently, under the two constraints 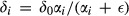 and 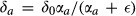. In other words, the pathogen can adjust its virulence to the host's immune response. This can be described as a form of phenotypic plasticity, even though the level of virulence 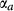 is determined genetically, because the timing of the switch follows the host's activation of its immune response. As mentioned earlier, this differs from the approach followed by Taylor *et al*. (2006), who allowed the level of virulence to respond to the host's phenotype, but only at the onset of infection.

The pathogen's ESS is determined by maximizing the basic reproductive ratio 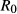; indeed, it can be shown that, in the absence of within-host competition, a mutant's fitness is independent of the genotype of the resident pathogen. The derivation of the evolutionarily stable levels of virulence can be found in Appendix S3. In the Results section below, I present a selection of numerical applications. In some cases (for example, high values of *μ* and 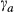), the ESS actually corresponds to a value of 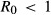, meaning that the pathogen cannot persist in the host population; in all the examples shown below, I checked the stability of the host–pathogen association.

### Host evolution

Whereas previous models for host–pathogen coevolution have considered the evolution of host defensive traits such as susceptibility to infection (*β*) or recovery rate (*γ*), here I assume that these traits are fixed; instead, I focus on the evolution of the rate of activation of the immune response (*μ*). The delay in mounting an immune response is constrained by factors including the recruitment of immune cells or the production of antimicrobial molecules. It is reasonable to assume that speeding up this process would incur costs, in terms of resource allocation and, possibly, autoimmune disorders (a more reactive immune system can be more prone to react to self). In this context, I consider that mutations that cause an increase in the activation rate *μ* will also result in a higher death rate *m*. In Appendix S4, I present analyses and results (very similar to those shown below) based on a reduction in fecundity rather than survival. Note that the cost is assumed to be constitutive, meaning that hosts with a higher activation rate will have a shorter lifespan even in the absence of pathogens; this ensures that, in the latter case, natural selection does favour hosts with no immune response (*μ* = 0). In the following, I present analyses and results with a simple linear relationship between activation rate and mortality, namely 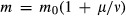, where 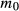 and *ν* are positive parameters (*ν* being the rate of activation that results in a two-fold increase in mortality). Alternative nonlinear cost functions give similar results, as shown in Appendix S4.

Evolutionary analyses were based on adaptive dynamics (see Appendix S4 for full details). First, I extended equations (1) to two competing host genotypes – a resident with activation rate 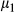 and a mutant with activation rate 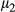. The ability of the mutant to invade was determined numerically by calculating next-generation matrices (Hurford *et al*., 2010). I used pairwise invasion plots to identify singular points and calculated the ES activation rates using a numerical optimization algorithm. All analyses were performed with Mathematica 8 (Wolfram Research Inc., Champaign, IL, USA); the code is available upon request.

Finally, I allowed the host and pathogen to coevolve, by embedding the expressions for the pathogens ES virulence and infectivity within the host's evolutionary algorithm (see Appendix S5). This effectively assumes that whenever a mutant host appears, it is faced with an endemic pathogen adapted to the resident host.

## Results

### Pathogen evolution

**Figure 2:**
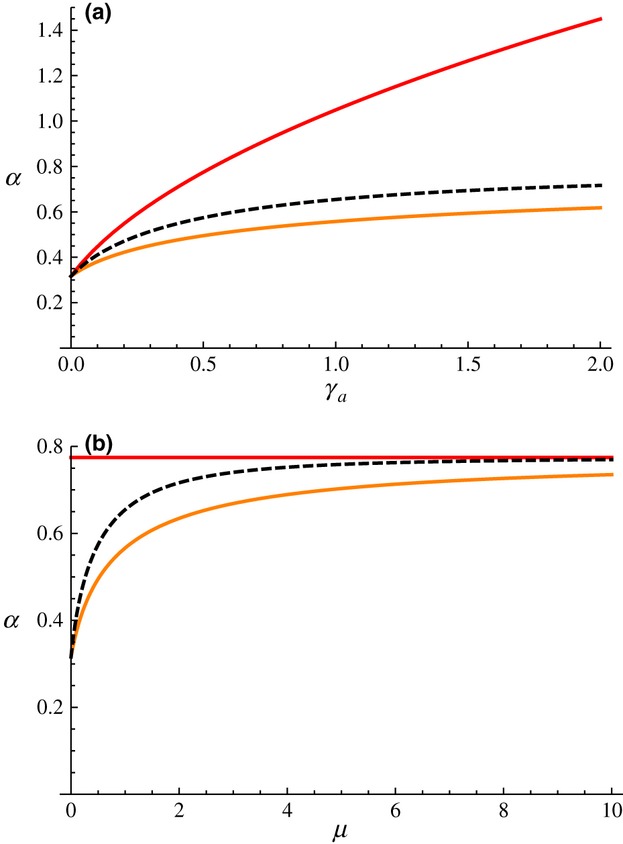
Pathogen's ES level of virulence, plotted against (a) the host's recovery rate 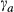 and (b) the rate of activation of the immune response *μ*. The dashed black line shows the ES virulence 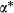 of nonplastic pathogens, whereas the amber and red lines show the respective ES levels 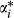 and 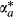 for plastic pathogens. Other parameter values: 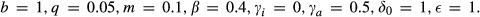

The first question I sought to address was how a pathogen would adapt to a host with a delayed immune response. Assuming a trade-off between virulence and infectivity, mathematical and numerical analyses reveal that a plastic strategy, with different levels of virulence before and after immune activation, is evolutionarily stable (Fig. 2a). The ES level of virulence following activation of the host's immune response is given by the simple expression 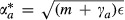. Thus, virulence is higher in hosts with a shorter lifespan or a stronger immune response, but it is independent of the basal clearance rate (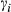) and the rate of immune activation. In the extreme case where the host does not mount an immune response (*μ* = 0), 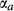 is no longer under selection because it is not expressed. The expression for the ES virulence during the initial phase, 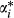, is more complicated and depends on *m*, *μ*, 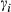, 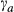 and *ε*. Numerically, I checked that 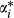 increases with each of these parameters (Fig. 2 and Appendix S3), as one could expect considering the negative effect of these parameters on the average duration of infection.

Figure 2 illustrates an important pattern: for a plastic pathogen, the ES virulence in the second phase is higher than the ES virulence before activation of the host's immune response (which is a direct consequence of the higher recovery rate), whereas the ES virulence of a nonplastic pathogen lies in-between 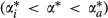. The position of the nonplastic ESS on Fig. 2b can be understood intuitively by noting that the relative contribution of the second phase of infection to the pathogen's total reproductive ratio is proportional to the rate of activation *μ* (see Appendix S2). Thus, for low values of *μ*, nonplastic pathogens behave like plastic pathogens during the first phase; conversely, with high values of *μ*, they behave like plastic pathogens during the second phase. Although the increase in virulence by plastic pathogens is driven by maximization of the pathogen's reproductive success, from the host's perspective, this strategy could be seen as a form of retaliation against the activation of its immune response. These results remain true if the recovery rate before immune activation is positive and lower than the post-activation rate.

### Host evolution

In this section, I investigate the evolution of the host's rate of immune activation, ignoring pathogen evolution; coevolution will be the subject of the next section. I begin with the case of a nonplastic pathogen, asking how immunological and epidemiological parameters affect selection on immune activation, before considering the effects of a plastic pathogen.

**Figure 3:**
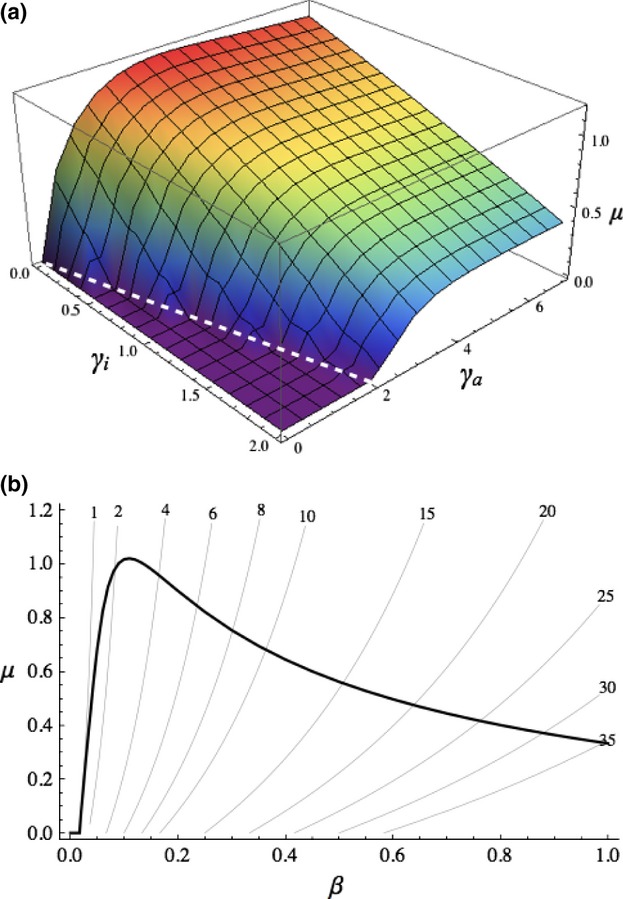
Host's ES rate of activation *μ* plotted against (a) recovery rates 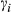 and 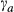 and (b) susceptibility to infection *β*. In both cases, the pathogen was nonplastic 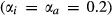. (a) The white dashed line indicates 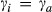. (b) Thin lines with numbered labels are 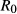 isoclines. Parameter values as in Fig. 2, except (a) *β* = 0.2, (b) 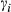 = 0 and 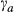 = 1.

The evolutionary responses to changes in the efficacy of host defences exhibit some noteworthy patterns, illustrated on Fig. 3 with a nonplastic pathogen (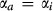). In this case, as may be expected, a nonzero activation rate can only evolve if the immune response increases the recovery rate, that is, 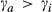 (Fig. 3a). Furthermore, whereas an increase in the baseline recovery rate 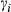 always selects for slower activation, the response to changes in 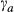 is not monotonic: *μ* initially increases with 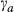 from low values until it reaches a maximum and then slowly decreases. Variations in host susceptibility to infection (*β*) produce a similar nonmonotonic evolutionary response (Fig. 3b).

The explanation for these nonmonotonic responses lies in ecological feedbacks in the evolution of host defences (Boots *et al*., 2009). In the present scenario, when 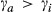, hosts with faster activation of their immune response are effectively more resistant to infection. As shown on Fig. 3, hosts with very efficient defences (low values of *β* or high values of 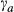) evolve slower immune activation than hosts with less efficient defences, thus keeping the pathogen's reproductive ratio 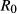 above 1 (see contours on Fig. 3b). At the other end of the spectrum, weak immune defences (high values of *β* or low values of 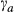) result in very high values of 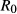, hence high prevalence of infection. In these conditions, even resistant hosts who clear infection rapidly get immediately reinfected, thus reducing the benefit of immune system activation. As a consequence, increasing the value of *β* or decreasing the value of 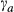 selects for lower activation rates *μ*.

**Figure 4:**
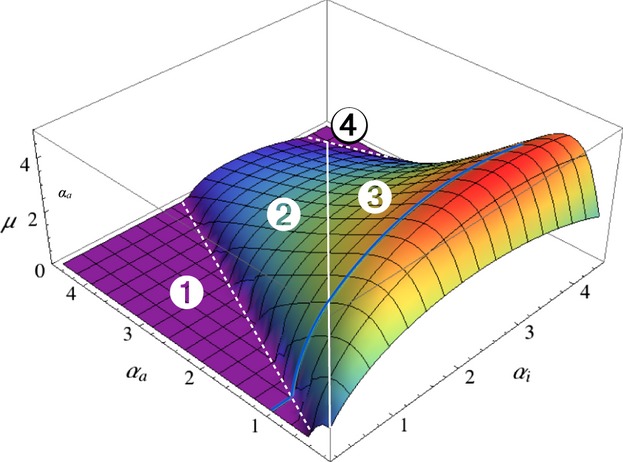
Host's ES rate of activation *μ* plotted against virulence levels 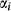 and 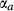. To allow comparison with the coevolutionary model, infectivity parameters vary along virulence levels, following 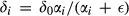 and 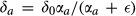. A similar graph was obtained when infectivity levels were kept constant instead (Appendix S4). The solid white line indicates where 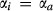; white dashed line delineate the region where *μ* > 0; white discs labelled 1–4 apply to regions separated by these three white lines. The blue line shows the pathogen's ESS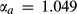 (see Fig. 5). Parameter values as in Fig. 3.

Let us now consider the effects of the pathogen's phenotypic plasticity. Fig. 4 is split into four regions (labelled from 1 to 4 and bounded by white lines) to illustrate the effects of pathogen plasticity; the solid white line that runs through the diagonal represents nonplastic pathogens 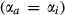. In region 1, virulence during the active immunity phase is at least twice as high as during the initial phase: this selects for hosts who never activate their immune response (*μ* = 0). In region 2, the increase in virulence following activation is less than two-fold, and this selects for relatively slow immune activation. As could be expected, faster activation evolves if virulence is lower after immune activation (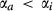, region 3). However, the latter scenario would not be expected to occur if pathogen evolution was taken into account, as we saw in the previous section that an efficient immune response 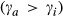 selects for an increase in virulence 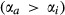. Finally, the ES activation rate decreases when either level of virulence reaches sufficiently high levels, up to the point where hosts no longer activate their immune response (region 4). This nonmonotonic response to changes in virulence is another example of the ecological feedbacks described above and is a hallmark of evolutionary models for host defences against pathogens (Gandon *et al*., 2002; Restif & Koella, 2004; Carval & Ferrière, 2010).

### Coevolution

**Figure 5:**
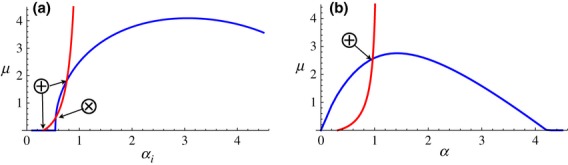
Host's ES rate of activation *μ* plotted against virulence levels: (a) 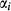 for a plastic pathogen and (b) *α* for a nonplastic pathogen. Blue lines show the host's ES rate of immune activation and red lines the pathogen's ES level of virulence. ⊕ indicates a coevolutionarily stable strategy and ⊗ a coevolutionary repeller. Parameter values as in Fig. 4.

By combining analyses of separate evolution of the host (Fig. 4) and pathogen (Fig. 2b), it is possible to predict the outcome of coevolution. As explained above, the ES virulence during the active immunity phase 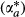 does not depend on the value of the activation rate *μ*. Hence, the blue line in Fig. 4 represents variations in the host's ES activation rate against a gradient of 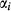 when 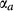 is set to the ES value (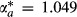 for the set of parameter values chosen). Conversely, the ES virulence 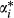 can be plotted against the activation rate *μ* (Fig. 2b and red line on Fig. 5a). Where the two lines intersect lies a coevolutionary equilibrium point. The stability of such points can be assessed easily using pairwise invasion plots (see Appendix S5). Thus, as shown in Fig. 5a, a pathogen with plastic virulence can give rise to two coevolutionarily stable strategies (CoESS, marked with ⊕ signs): one with a relatively rapid immune activation and the other with no activation (*μ* = 0) and a lower pathogen virulence. They are separated by a 'coevolutionary repeller' (marked with a ⊗ sign), that is, a state which is evolutionarily stable (neither the host nor the pathogen can be invaded by any rare mutant) but unstable by convergence (it cannot evolve by small mutations from a different combination of genotypes). The outcome of coevolutionary dynamics in a particular population would depend on the initial genotypes present and the mutation regime (which I will not study here). In contrast, with a nonplastic pathogen there can only be one CoESS, with intermediate levels of virulence and activation (Fig. 5b).

**Figure 6:**
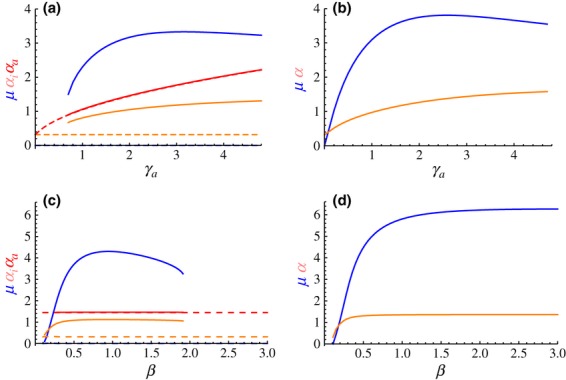
Coevolutionary stable strategies plotted against (a, b) recovery rate 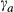 and (c, d) susceptibility to infection *β*. Blue lines show the host's immune activation rate *μ*. (a, c) Plastic pathogen: amber and red lines show the pathogen's respective virulence levels 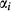 and 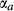; dashed lines show the lower CoESS, with *μ* = 0 (see Fig. 5a). (b, d) Nonplastic pathogen: amber lines show the pathogen's virulence level *α*.

I now ask how the strength of the host defences affects the coevolutionary equilibrium. We saw earlier that a stronger immune response tends to select for higher virulence (Fig. 2a) and that both factors have nonmonotonic effects on host evolution (Figs 3 and 4). Although coevolution with a nonplastic pathogen reproduces these patterns (Fig. 6b,d), a pathogen with plastic virulence creates more complex outcomes, with one or two CoESS. The first CoESS, with no immune activation and low virulence, always exists (dashed lines on Fig. 6a,c). The second CoESS, with a positive rate of activation and higher levels of virulence (solid lines on Fig. 6a,c), vanishes if host defences are not strong enough: low values of 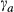 (Fig. 6a) generate a low benefit for mounting a costly immune response, whereas high values of susceptibility *β* (Fig. 6c) result in a high probability of reinfection.

The contrast between plastic and nonplastic virulence is striking (left vs. right panels on Fig. 6). Even a moderate change in virulence between the two phases of infection can select for hosts that never activate their acquired immune response (dashed lines on Fig. 6a,c). When it exists, the alternative CoESS (solid lines on Fig. 6a,c) has a rate of activation lower than that of the CoESS with nonplastic virulence (Fig. 6b,d). It is as if the threat of an increase in virulence was sufficient to force the host to delay (or even suppress) the activation of its immune response.

The case of the CoESS with no immune activation (dashed lines on Fig. 6a,c) deserves further comments. In this case, the system appears to work as a simpler model with a single stage of infection. However, in theory, both the host and the parasite have, encoded in their genomes, the potential for a second stage of infection with both higher recovery rate and higher virulence. The dashed red lines on Fig. 6a,c show this theoretical level of virulence. As explained in the previous section, virulence 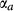 is no longer submitted to selection; the dashed line indicates the value (given by 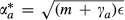) that would be selected if the host mounted an immune response, regardless of the corresponding rate of activation. However, if further mutations were to remove the pathogen's ability to adjust its level of virulence during the second phase of infection, then the system would evolve towards the single CoESS shown on Fig. 6b,d, with a substantially higher level of virulence and rapid activation of the host's acquired immune response. In other words, the phenotypic plasticity of the pathogen can maintain the system in a stable, commensal relationship, preventing an escalating arms race with the (unrealized) threat of collateral damage. If the infection can be cleared without activation of the immune response (i.e. with high values of 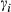, as shown in the Appendix S5), the CoESS with no activation, although still present, cannot be reached through small mutations – it is no longer convergence stable, thus forming a singularity known as 'Garden of Eden' (de Mazancourt & Dieckmann, 2004).

## Discussion

### Ecology and evolution of immune defences

The model presented here extends previous studies of host–pathogen coevolution (van Baalen, 1998; Day & Burns, 2003; Restif & Koella, 2003) within the framework of adaptive dynamics, adding an important feature common to many host–pathogen systems: variations in both the efficiency of the immune response and the level of virulence during the course of infection. Like those earlier models, it highlights the role played by population dynamics in shaping the pressures of selection on the host's immune defences (Boots *et al*., 2009). Whereas the evolutionary implications of the physiological costs of immunity have started to be considered (Graham *et al*., 2005; Long & Graham, 2011), the ecological dimension has been largely overlooked (Duffy & Forde, 2009). The focus of most experimental studies of host–pathogen coevolution has been on antagonistic arms races (Buckling & Rainey, 2002; Allen *et al*., 2004; Arnaud *et al*., 2007), effectively assuming that natural selection should always favour more resistant hosts. Although some coevolutionary studies have measured the cost of resistance (Forde *et al*., 2008; Morgan *et al*., 2009; Schulte *et al*., 2010), its benefits have rarely been measured in a context relevant to natural selection. Novel experimental approaches need to be developed to assess the importance of these ecological feedbacks. Meanwhile, new theoretical models should account for more realistic features to generate testable predictions; this is the main motivation for the present study. Progress has been made in modelling the selective pressures caused by complex immune responses on pathogens within hosts (Fenton *et al*., 2006; Alizon & Boldin, 2010), but integrating these with host population dynamics remains an important challenge (Alizon *et al*., 2011).

The main prediction of the model presented here is that pathogen phenotypic plasticity can promote the evolution of commensal associations, characterized by delayed activation of the immune response and low virulence. This result is in qualitative agreement with that of Taylor *et al*. (2006), although they modelled phenotypic plasticity with a continuous reaction norm, allowing phenotypes to vary through an iterative negotiation process. The approach I used eliminates the issue of iterative information exchange between the host and the symbiont: here, the only assumption is that the pathogen can switch between two predetermined levels of virulence as soon as the host activates its immune response; there is no exchange of information on the levels of virulence or the strength of the immune response, all of which are genetically determined.

This modelling framework applies to any form of delayed up-regulation of immune defences, whether infection can be cleared or not before this activation. For example, in vertebrate hosts, the so-called adaptive immune response is usually triggered at a later stage of infection, under strict control of the innate immune system. Even though the model presented here lacks much of the complexity of the adaptive immune system, it could provide a starting point to incorporate more immunological realism into eco-evolutionary models. An additional feature of the adaptive immune system is a form of memory that enables a rapid response upon re-exposure to the same pathogen. I have considered this property in an extension of the model (presented in the online appendix), which assumes that previously infected hosts can mount an acquired immune response without delay following reinfection, provided they have mounted one during the first infection. Although this is expected to increase the benefits of an early activation of the immune system, the evolutionary predictions remain unchanged (see additional results in the online appendix). This is in large part due to the fact that the anamnestic response does not affect the pathogen's evolution in this model: in the absence of antigenic variation, all pathogen strains are equally infectious to immune hosts.

The prediction that hosts could evolve to down-regulate the activation of their immune response raises questions about the case of co-infection. In this model, pathogens compete for access to susceptible hosts, but it is well established that competitive or cooperative interactions between pathogens inside a host can affect the evolution of virulence in different ways (Alizon & Lion, 2011). How hosts evolve in the presence of co-infecting pathogens will depend on the specificity of the immune detection and response. For example, it has been suggested that diversity of major histocompatibility complex genes could be maintained due to increased resistance of heterozygotes to multiple parasitic infections (Oliver *et al*., 2009). Yet, there is currently a lack of models considering host evolution in response to co-infecting pathogens or parasites.

### Mafia behaviour?

The mafia analogy was coined by Soler *et al*. (1995) to explain why certain bird species parasitized by cuckoos raise the alien offspring, whereas other species can identify and eject the parasites eggs. Their study experimentally tested Zahavi (1979) hypothesis that predatory retaliation by cuckoos would dissuade host birds to eject eggs. Mathematical models have been developed to explore the conditions under which such behaviours could evolve (Pagel *et al*., 1998; Robert *et al*., 1999), and experimental studies have further demonstrated the existence of this phenomenon in different brood parasite species (Soler *et al*., 1998; Hoover & Robinson, 2007). Although it has been suggested that the mafia analogy might apply to a wide range of parasites known to manipulate the behaviour of their hosts (Ponton *et al*., 2006), evidence remains scarce.

In the present study, 'rejection' is modelled as the activation of the immune response and 'retaliation' as an increase in virulence. There is no behavioural change as such but hardwired plasticity in phenotypes. Although repeated interactions do occur in the form of reinfection following parasite clearance, neither actor can change its strategy. In addition, virulence as a form of retaliation is a much more radical option because it kills both the host and the pathogen, preventing any opportunity to learn from one's mistake. Unexpectedly, the model predicts two possible coevolutionary outcomes, both of which bear similarities with the mafia analogy. According to the first scenario, which occurs when host defences are strong enough, 'retaliation' (i.e. plastic increase in virulence) selects for hosts with slower immune activation and for pathogens with reduced virulence in the initial phase and higher virulence in the second phase (compared with the ESS of virulence in nonplastic pathogens; solid lines in Fig. 6). In this scenario, the host and the pathogen seem to have found a sort of compromise, but 'rejection' and 'retaliation' still occur. This outcome only happens if the immune defence is strong enough, so that the host still has an incentive to activate its response. In contrast, under the second scenario, which is always stable, hosts do not activate their immune response at all, so retaliation is not implemented (dashed lines in Fig. 6): it is as if the threat of increased virulence was sufficient to keep the host in a tolerant state. The resulting level of virulence is much lower, so that the association is actually closer to commensalism. Importantly, infection still carries a cost to the host. The two outcomes coexist over a large set of parameter values, and they are both globally evolutionarily stable (in particular neither can invade the other) and both are convergence stable (they can evolve through a series of small mutations).

Anthropomorphic analogies are commonly used in evolutionary biology but their limitations should always be clearly stated. Here, the increase in virulence following the activation of an immune response may be seen as a form of 'retaliation' from the host's perspective only. What actually selected for this increase was the lower environmental quality experienced by the pathogen. Yet the similarity between the host's evolution in this model with that of animals exposed to retaliatory brood parasites is striking.

### Conclusions

Beyond the thought-provoking analogy, this study provides a new hypothesis for the maintenance of a commensal association. Whereas host–pathogen coevolution is traditionally expected to lead to an escalating arms race, I have demonstrated that, under simple assumptions, a low-virulence, low-resistance equilibrium can be evolutionarily stable. The only addition to previous coevolutionary models is a delay in the activation of the host's immune response, to which the pathogen can respond by adjusting its virulence. Stepwise changes in immune responses and pathogen virulence have been documented, for example during *Salmonella* infection (Mastroeni *et al*., 2009). To validate the model's assumption in a particular host–pathogen system, one would need to measure changes in virulence of a given pathogen genotype in host genotypes differing in their immune responses. The adaptive dynamic framework used here provides a simple way to help understand the impact of specific mechanisms on the selective pressures shaping the evolution of the host and pathogen. Although this approach is based on pairwise competition between genotypes, recent theoretical developments offer possible ways to account for the whole mutation-selection process (Gandon & Day, 2009) or quantitative phenotypic plasticity (Taylor *et al*., 2006).

Thirty years after May & Anderson's (1983) seminal presentation of a theoretical framework for the study of host–pathogen coevolution, and despite a flourishing legacy in evolutionary ecology, its influence in the field of immunology has remained marginal (Schneider & Ayres, 2008). By adding assumptions relevant to human and animal infections, it is hoped that this study will contribute to a more fruitful dialogue between disciplines.

## Supporting Information

Appendix S1 Model definitions. **Appendix S2** Pathogen basic reproductive ratio. **Appendix S3** Pathogen evolution. **Appendix S4** Host evolution. **Appendix S5** Coevolution.
