# Supplementary material for: An offer you cannot refuse: down-regulation of immunity in response to a pathogen's retaliation threat
Source: J Evol Biol. 2013 Aug 9;26(9):2021–30. doi: 10.1111/jeb.12209 (PMC4274018; doi:10.1111/jeb.12209)
Supplement: Appendix S1 — Model definitions. Appendix S2 Pathogen basic reproductive ratio. Appendix S3 Pathogen evolution. Appendix S4 Host evolution. Appendix S5 Coevolution. [file jeb0026-2021-sd1.pdf]

An offer you cannot refuse: down-regulation of immunity in response to a pathogen's retaliation threat.

Electronic Supplementary Material

Olivier Restif

Contents

1. Model definitions ***2***

2. Pathogen basic reproductive ratio ***4***

3. Pathogen evolution ***5***

a. Non-plastic virulence 5

b. Plastic virulence 6

c. Numerical results 7

4. Host evolution ***8***

a. SIAS model 8

b. Supplementary result 12

c. SIARA model 12

5. Coevolution ***16***

a. SIAS model, plastic virulence 16

b. SIAS model, fixed virulence 17

c. SIARA model, plastic virulence 17

d. SIARA model, fixed virulence 18

Bibliography ***19***

1. Model definitions

The baseline population dynamic model as presented in the main text (Fig. 1) is described by the following set of differential equations:

$$\begin{cases} \frac{dS}{dt} = bN(1-qN) - \beta S(\delta_i I + \delta_a A) - mS + \gamma_i I + \gamma_a A \\ \frac{dI}{dt} = \beta S(\delta_i I + \delta_a A) - (m + \alpha_i + \gamma_i + \mu) I \\ \frac{dA}{dt} = \mu I - (m + \alpha_a + \gamma_a) A \end{cases} \quad \text{(S1)}$$

An extended model describing acquired immunity with memory (Fig. S1) is given by the following set of differential equations:

$$\begin{cases} \frac{dS}{dt} = bN(1-qN) - \beta S(\delta_i I + \delta_a A) - mS + \gamma_i I \\ \frac{dI}{dt} = \beta S(\delta_i I + \delta_a A) - (m + \alpha_i + \gamma_i + \mu) I \\ \frac{dA}{dt} = \mu I + \beta R(\delta_i I + \delta_a A) - (m + \alpha_a + \gamma_a) A \\ \frac{dR}{dt} = \gamma_a A - \beta R(\delta_i I + \delta_a A) - mR \end{cases} \quad \text{(S2)}$$

All symbols are defined in Table S1. In the following I refer to model (S1) as the SIAS model, and (s2) as the SIARA model.

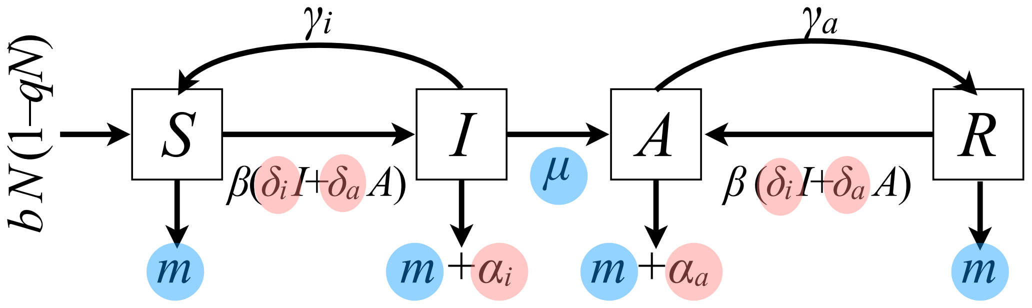

Figure S1. Schematic of the extended population dynamic model with immune memory. Hosts that have mounted an immune response (A) move into the (R) compartment following recovery; upon reinfection, they mount an immune response immediately.

Table S1. List of symbols used in the models.

Symbol	Description	Default value
S	Density of susceptible hosts	
I	Density of infected hosts in the initial phase	
A	Density of infected hosts with an active immune response	
R	Density of recovered hosts with immune memory (extended model only)	
N	Total host population density	
b	Maximal birth rate per capita	1
q	Susceptibility to crowding (density-dependent reduction in birth rate)	0.05
m	Host death rate per capita	0.1
β	Host susceptibility to infection	0.2
δ_i	Infectivity of hosts before immune activation	1
δ_a	Infectivity of hosts after immune activation	1
α_i	Additional death rate (virulence) of hosts before immune activation	0.4
α_a	Additional death rate (virulence) of hosts following immune activation	0.4
γ_i	Baseline recovery rate of hosts without immune response	0
γ_a	Recovery rate with an active immune response	1
μ	Rate of activation of the immune response	1
R_0	Basic reproductive ratio of the pathogen	4
K	Carrying capacity[1] of the host population	18

[1] The carrying capacity is defined as the stable equilibrium of the host population in the absence of infection. For both models it is equal to $K = \dfrac{b - m}{bq}$.

2. Pathogen basic reproductive ratio

In order to determine the pathogen's basic reproductive ratio, I used next generation matrices as described by Hurford et al. [1]. While there are simpler heuristics to derive the expression of R_0, the use of the next-generation theorem is more systematic and can also be used to determine invasion criteria for host and pathogen evolution. In brief, I re-write the system of differential equations in vectorial form $d\mathbf{x}/dt = \mathbf{A}\mathbf{x}$, where $\mathbf{x}(t)$ is the vector of state variables of the system and $\mathbf{A}$ is a matrix of constant coefficients. The next-generation theorem states that, if $\mathbf{A}$ can be written as $\mathbf{F}-\mathbf{V}$ where $\mathbf{F}\geq 0$, $\mathbf{V}^{-1}\geq 0$ and if all the eigenvalues of $-\mathbf{V}$ have negative real parts, then all the eigenvalues of A have negative real parts if and only if all the eigenvalues of $\mathbf{F}\mathbf{V}^{-1}$ lie within the unit circle [1].
In practice, "**F** is a matrix which gives the rate at which new individuals appear in class j, per individual of type i. The matrix **V** describes the movement of existing individuals among the different classes, as well as the loss of these individuals. […] Hence, $\mathbf{F}\mathbf{V}^{-1}$ is sometimes referred to as the next-generation matrix. Moreover, $\rho(\mathbf{F}\mathbf{V}^{-1}) = R_0$, which has an interpretation as the expected lifetime reproductive output of a newborn individual." [1]
To calculate R_0 for the pathogen in either model, I assume that the host population is fully susceptible and at the carrying capacity K. The state vector for the pathogen is $\mathbf{x} = (I \quad A)^{\mathrm{T}}$, the reproduction matrix is

$$\mathbf{F} = \begin{pmatrix} \beta\delta_i K & \beta\delta_a K \\ 0 & 0 \end{pmatrix}$$

and the transition matrix is

$$\mathbf{V} = \begin{pmatrix} m+\alpha_i+\gamma_i+\mu & 0 \\ -\mu & m+\alpha_a+\gamma_a \end{pmatrix}.$$

The dominant eigenvalue of $\mathbf{F}\mathbf{V}^{-1}$ is then $R_0 = \dfrac{\beta K\left[\delta_i\left(m+\alpha_a+\gamma_a\right)+\delta_a\mu\right]}{\left(m+\alpha_i+\gamma_i+\mu\right)\left(m+\alpha_a+\gamma_a\right)}$ (S3).

Note that the same expression could be obtained by reasoning from first principles, writing R_0 as the sum of reproductive ratios during the two phases of infection:

$$R_0 = \frac{\beta\delta_i K}{m+\alpha_i+\gamma_i+\mu} + \left(\frac{\mu}{m+\alpha_i+\gamma_i+\mu}\right)\frac{\beta\delta_a K}{m+\alpha_a+\gamma_a}$$

where the factor between brackets is the probability of reaching the second phase of infection.

Even though the full expressions of the equilibrium points of either system cannot be obtained analytically, it can be shown by solving system (S1) that, the endemic equilibrium (S^*, I^*, A^*) must satisfy the following relations:

$$S^* = \frac{K}{R_0},\ A^* = \frac{\mu I^*}{m+\alpha_a+\gamma_a}.$$

3. Pathogen evolution

To study pathogen evolution, I extend equations (S1) and (S2) to two strains of pathogens that compete for infection of susceptible hosts (S and R), assuming that currently infected hosts (I or A) cannot be reinfected. In addition, the two pathogen strains only differ by the values of virulence (α_i and α_a) and infectivity (δ_i and δ_a) and are antigenically identical, so that hosts are equally susceptible to both strains.

Labelling the two strains with subscripts 1 and 2, the SIAS model can be written as:

$$\begin{cases} \frac{dS}{dt} = bN(1-qN) - \beta S(\delta_{i,1} I_1 + \delta_{a,1} A_1 + \delta_{i,2} I_2 + \delta_{a,2} A_2) - mS + \gamma_i I_1 + \gamma_a A_1 + \gamma_i I_2 + \gamma_a A_2 \\ \frac{dI_1}{dt} = \beta S(\delta_{i,1} I_1 + \delta_{a,1} A_1) - (m + \alpha_{i,1} + \gamma_i + \mu) I_1 \\ \frac{dA_1}{dt} = \mu I_1 - (m + \alpha_{a,1} + \gamma_a) A_1 \\ \frac{dI_2}{dt} = \beta S(\delta_{i,2} I_2 + \delta_{a,2} A_2) - (m + \alpha_{i,2} + \gamma_i + \mu) I_2 \\ \frac{dA_2}{dt} = \mu I_2 - (m + \alpha_{a,2} + \gamma_a) A_2 \end{cases}$$

Assuming that both strains have basic reproductive ratios $R_{0,1}$ and $R_{0,2}$ greater than unity, I consider the scenario where strain 1 is initially present and has reached endemic equilibrium (S^*, I_1^*, A_1^*) and strain 2 is introduced at a very low prevalence.
Using the next generation theorem as before, the mutant's fitness can be written as:

$$R_{m,2} = \frac{\beta S^* \left[\delta_{i,2}(m + \alpha_{a,2} + \gamma_a) + \delta_{a,2}\mu\right]}{(m + \alpha_{i,2} + \gamma_i + \mu)(m + \alpha_{a,2} + \gamma_a)} = \frac{S^*}{K} R_{0,2} = \frac{R_{0,2}}{R_{0,1}}$$

Hence the mutant will be able to invade if and only if its basic reproductive ratio is larger than that of the resident strain. The same analysis can be done easily with the SIARA model and leads to the same conclusion.

As explained in the main text, I chose to impose a 'classical' constraint on pathogen evolution by assuming that virulence α and infectivity δ are positively linked. In a single-stage infection (here when $\mu = 0$), if infectivity increases less than linearly with virulence, there is a single phenotype that maximises R_0 and it is therefore an ESS [2, 3]. Here I consider two cases, depending on whether the pathogen has a plastic response or not.

a. Non-plastic virulence

First, if the pathogen cannot change its virulence, let $\alpha_i = \alpha_a = \alpha$ and $\delta_i = \delta_a = \delta(\alpha)$. Then the first order condition for α to be an ESS is:

$$\frac{\delta'(\alpha)}{\delta(\alpha)} = \frac{1}{\alpha + m + \gamma_i + \mu} + \frac{\mu}{(\alpha + m + \gamma_a)(\alpha + m + \gamma_a + \mu)} \tag{S4}$$

Using the function $\delta(\alpha) = \delta_0 \frac{\alpha}{\alpha + \varepsilon}$, equation (S4) becomes a quartic polynomial. While it cannot be solved analytically, it is possible to prove that equation (S4) has exactly one positive root α^* with $0 < \alpha^* < \sqrt{\varepsilon(m + \mu + \gamma_i)}$.

Proof: Let $A = m + \gamma_i + \mu$, $B = m + \gamma_a$, $C = m + \gamma_a + \mu$. Equation S4 can then be re-written as:

$$(\alpha + B)(\alpha + C)\left(\alpha + \sqrt{\varepsilon A}\right)\left(\alpha - \sqrt{\varepsilon A}\right) = -\mu\alpha(\alpha + \varepsilon)(\alpha + A)$$

Let $F_4(\alpha)$ be the quartic polynomial on the left-hand side and $F_3(\alpha)$ the cubic polynomial on the right-hand side. Since the two polynomials have all but one negative roots, they are monotonic over the positive domain. For positive values of α, $F_4(\alpha)$ is an increasing function, negative between 0 and $\sqrt{\varepsilon A}$ and positive beyond, while $F_3(\alpha)$ is a decreasing function with $F_3(0) = 0 > F_4(0)$. Hence there is a unique positive value of α such that $F_4(\alpha) = F_3(\alpha)$ and it is lower than $\sqrt{\varepsilon A}$. QED.

The second-order condition $R_0''\left(\alpha^*\right) < 0$ boils down (after a few lines of tedious algebra) to $\gamma_a > \gamma_i$ which is the working assumption. I therefore conclude that a non-plastic pathogen has a single ESS α^*, which satisfies $0 < \alpha^* < \sqrt{\varepsilon(m + \mu + \gamma_i)}$.

b. Plastic virulence

If the pathogen can change its virulence during the second phase of infection, so that the values of α_i and α_a are evolving independently, then any candidate ESS has to be a solution of the following system of two equations:

$$\begin{cases} \delta_i'(\alpha_i) = \dfrac{R_0(\alpha_i, \alpha_a)}{\beta K} \\ \delta_a'(\alpha_a) = \dfrac{\delta_a(\alpha_a)}{\alpha_a + m + \gamma_a} \end{cases} \qquad \text{(S5)}$$

Using the function $\delta(\alpha) = \delta_0 \dfrac{\alpha}{\alpha + \varepsilon}$ for both δ_i and δ_a, (S5) has a unique solution:

$$\begin{cases} \alpha_i^* = \dfrac{-\mu\varepsilon\alpha_a^* + \sqrt{\varepsilon f(\alpha_a^*)\left[f(\alpha_a^*)(m + \gamma_i) + \mu\alpha_a^*(2m + \alpha_a^* + \gamma_i + \gamma_a) + \varepsilon\mu(m + \gamma_a) + \mu^2\alpha_a^*\right]}}{f(\alpha_a^*) + \mu\alpha_a^*} \\ \alpha_a^* = \sqrt{\varepsilon(m + \gamma_a)} \end{cases}$$

where $f(\alpha_a^*) = (\alpha_a^* + \varepsilon)(\alpha_a^* + m + \gamma_a)$

There remains to calculate the Hessian matrix of $R_0(\alpha_i, \alpha_a)$:

$$H(\alpha_i^*, \alpha_a^*) = \begin{pmatrix} \dfrac{\partial^2 R_0}{\partial \alpha_i^2}(\alpha_i^*, \alpha_a^*) & \dfrac{\partial^2 R_0}{\partial \alpha_i \partial \alpha_a}(\alpha_i^*, \alpha_a^*) \\ \dfrac{\partial^2 R_0}{\partial \alpha_a \partial \alpha_i}(\alpha_i^*, \alpha_a^*) & \dfrac{\partial^2 R_0}{\partial \alpha_a^2}(\alpha_i^*, \alpha_a^*) \end{pmatrix} = \begin{pmatrix} \dfrac{\beta K \delta''(\alpha_i^*)}{(m + \alpha_i^* + \gamma_i + \mu)} & 0 \\ 0 & \dfrac{\beta K \mu \delta''(\alpha_a^*)}{(m + \alpha_i^* + \gamma_i + \mu)(m + \alpha_a^* + \gamma_a)} \end{pmatrix}$$

Since δ is a concave function, the two terms on the diagonal are negative, hence (α_i^*, α_a^*) is a maximum of R_0, so it is an ESS.

c. Numerical results

Pathogen's ES levels of virulence plotted against the host's activation rate (μ), host mortality (m), and recovery rate before (γ_i) or after immune activation (γ_a). The dashed black line shows the ES virulence α^* of non-plastic pathogens, whereas the amber and red lines show the respective ES levels α_i^* and α_a^* for plastic pathogens. Same numerical values as on Fig. 2.

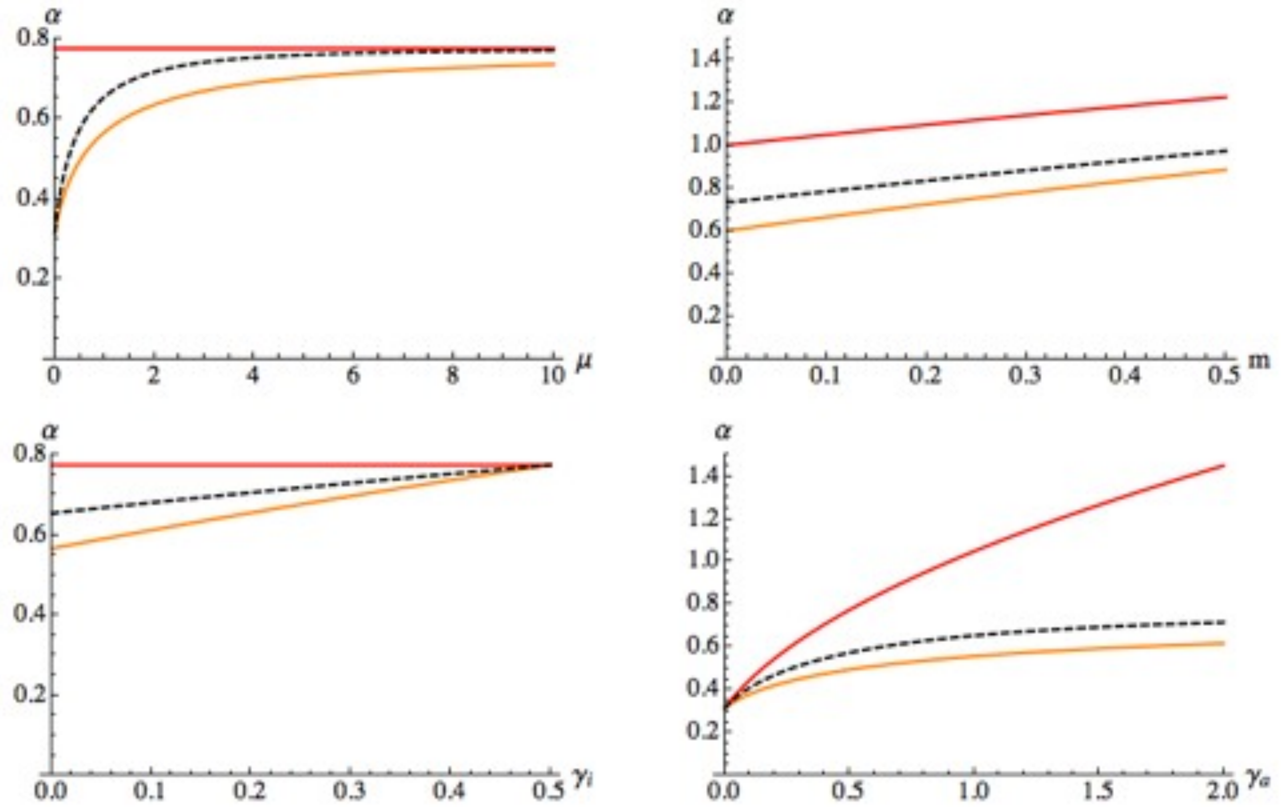

4. Host evolution

a. SIAS model

In this section I consider two competing host genotypes (labelled with subscripts 1 and 2) that can differ in three traits: immune activation rate μ, fecundity b and mortality m. There is a single strain of pathogen which does not evolve. The SIAS model can be written as:

$$\begin{cases} \frac{dS_1}{dt} = b_1\left(S_1 + I_1 + A_1\right)\left(1 - qN\right) - \beta S_1\left(\delta_i I_1 + \delta_a A_1 + \delta_i I_2 + \delta_a A_2\right) - m_1 S_1 + \gamma_i I_1 + \gamma_a A_1 \\ \frac{dI_1}{dt} = \beta S_1\left(\delta_i I_1 + \delta_a A_1 + \delta_i I_2 + \delta_a A_2\right) - \left(m_1 + \alpha_i + \gamma_i + \mu_1\right) I_1 \\ \frac{dA_1}{dt} = \mu_1 I_1 - \left(m_1 + \alpha_a + \gamma_a\right) A_1 \\ \frac{dS_2}{dt} = b_2\left(S_2 + I_2 + A_2\right)\left(1 - qN\right) - \beta S_2\left(\delta_i I_1 + \delta_a A_1 + \delta_i I_2 + \delta_a A_2\right) - m_2 S_2 + \gamma_i I_2 + \gamma_a A_2 \\ \frac{dI_2}{dt} = \beta S_2\left(\delta_i I_1 + \delta_a A_1 + \delta_i I_2 + \delta_a A_2\right) - \left(m_2 + \alpha_i + \gamma_i + \mu_2\right) I_2 \\ \frac{dA_2}{dt} = \mu_2 I_2 - \left(m_2 + \alpha_a + \gamma_a\right) A_2 \end{cases} \qquad \text{(S6)}$$

As with the pathogen, I follow Hurford et al.'s [1] next-generation matrix method to determine the conditions under which the mutant genotype 2 can invade the resident genotype 1. I assume that the system has reached its stable equilibrium $\left(S_1^*, I_1^*, A_1^*\right)$ in the absence of genotype 2 and that genotype 1 is such that the pathogen's basic reproductive ratio is greater than unity. From system (S6), I define the hosts' reproduction matrix **F** as:

$$\mathbf{F} = \left(1 - qN_1^*\right)\begin{pmatrix} b_1 & b_1 & b_1 & 0 & 0 & 0 \\ 0 & 0 & 0 & 0 & 0 & 0 \\ 0 & 0 & 0 & 0 & 0 & 0 \\ 0 & 0 & 0 & b_2 & b_2 & b_2 \\ 0 & 0 & 0 & 0 & 0 & 0 \\ 0 & 0 & 0 & 0 & 0 & 0 \end{pmatrix}$$

and the transition matrix **V** as:

$$\mathbf{V} = \begin{pmatrix} m_1 + \Lambda_1^* & -\gamma_i & -\gamma_a & 0 & 0 & 0 \\ -\Lambda_1^* & m_1 + \alpha_i + \gamma_i + \mu_1 & 0 & 0 & 0 & 0 \\ 0 & -\mu_1 & m_1 + \alpha_a + \gamma_a & 0 & 0 & 0 \\ 0 & 0 & 0 & m_2 + \Lambda_1^* & -\gamma_i & -\gamma_a \\ 0 & 0 & 0 & -\Lambda_1^* & m_2 + \alpha_i + \gamma_i + \mu_2 & 0 \\ 0 & 0 & 0 & 0 & -\mu_2 & m_2 + \alpha_a + \gamma_a \end{pmatrix}.$$

where $\Lambda_1^* = \beta\left[\delta_i I_1^* + \delta_a A_1^*\right]$ is the force of infection at equilibrium. It follows that the next-generation matrix $\mathbf{FV}^{-1}$ has the same zero elements as **F**, and therefore has two non-zero eigenvalues ω_1 and ω_2 which represent the respective relative fitnesses of genotypes 1 and 2:

$\omega_1 = 1$

$$\omega_2 = \frac{b_2\left(1-qN_1^*\right)\left[\left(m_2+\alpha_a+\gamma_a\right)\left(m_2+\alpha_i+\gamma_i+\Lambda_1^*\right)+\mu_2\left(m_2+\alpha_a+\gamma_a+\Lambda_1^*\right)\right]}{\left(m_2+\alpha_a+\gamma_a\right)\left[\alpha_i\Lambda_1^*+m_2\left(m_2+\alpha_i+\gamma_i+\Lambda_1^*\right)\right]+\mu_2\left[\alpha_a\Lambda_1^*+m_2\left(m_2+\alpha_a+\gamma_a+\Lambda_1^*\right)\right]} \quad \text{(S7).}$$

Strain 2 can invade if and only if $\omega_2 > 1$. Since the expressions for the equilibrium $\left(S_1^*, I_1^*, A_1^*\right)$ cannot be obtained analytically, invasion analyses have to be performed numerically.

In the absence of any constraints, natural selection would favour maximum values of μ and b and minimum values of m. In the following I assume that any increase in activation rate μ comes at a cost on either survival (increase in m) or fecundity (lower b). The following figures are Pairwise Invasibility Plots (PIP) where the value of the mutant's fitness ω_2 is colour-coded as a function of the resident's activation rate μ_1 on the horizontal axis and mutant's activation rate μ_2 on the vertical axis: black areas show combinations of genotypes that allow the mutant to invade ($\omega_2>1$). This technique enables the visualisation of evolutionary singularities as points where the boundary of a black area crosses the diagonal [4].

(i) Linear cost on mortality: $m = m_0\left(1+\mu/\nu\right)$. Numerical values as in Table S1 and m_0=0.1, ν=10.

$\gamma_i = 0, \gamma_a = 0.2$ | $\gamma_i = 0, \gamma_a = 1$ | $\gamma_i = 0.5, \gamma_a = 1$

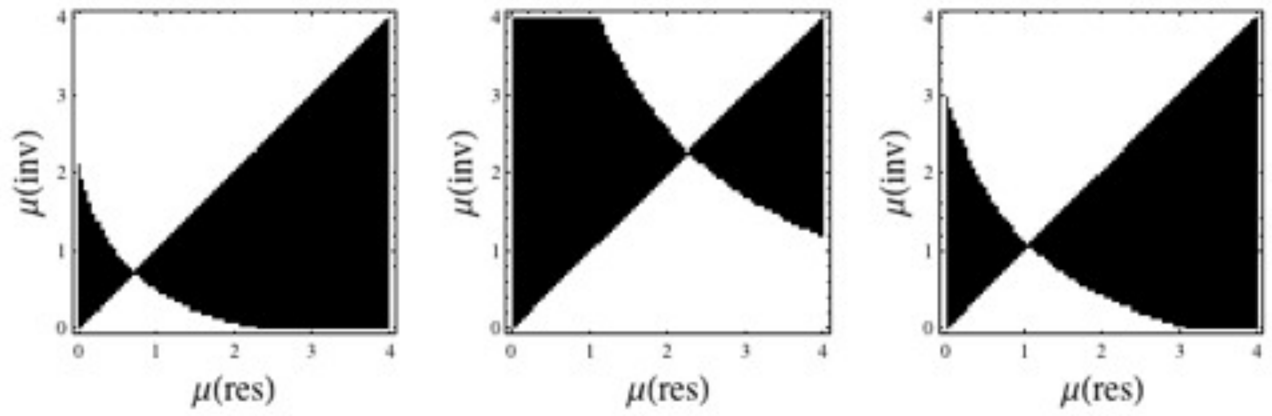

These graphs show a single ESS which is both evolutionarily stable (it cannot be invaded by any mutant) and convergent-stable (it can evolve through a series of small mutations from any other genotype). The ESS drops to zero when the effective benefit of mounting an immune defence is too low, for example if virulence is too high during the second phase of infection:

$\alpha_a = 0.57$ | $\alpha_a = 0.58$ | $\alpha_a = 0.59$

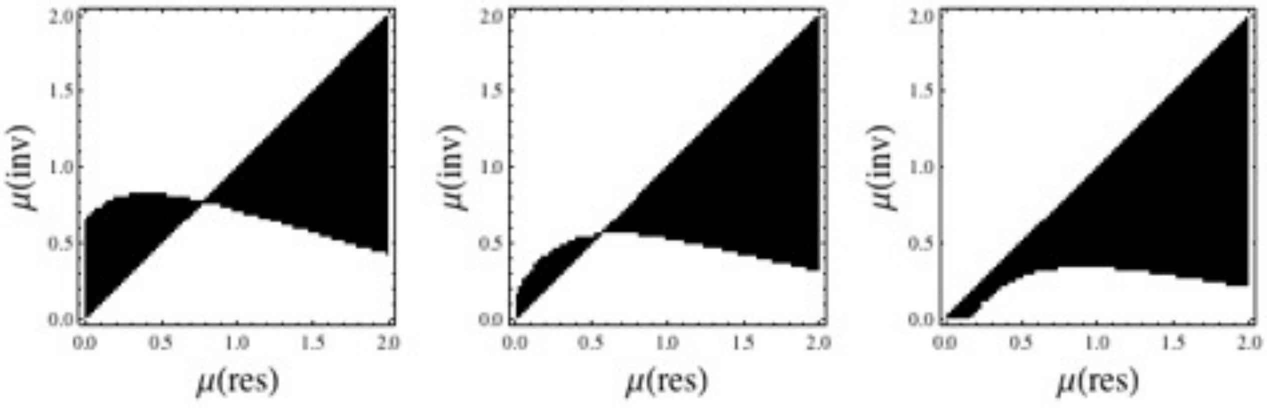

(ii) Quadratic cost on mortality: $m = m_0\left[1+(\mu/\nu)^2\right]$. Numerical values as in (i).

$\gamma_i = 0$, $\gamma_a = 0.2$ $\gamma_i = 0$, $\gamma_a = 1$ $\gamma_i = 0.5$, $\gamma_a = 1$

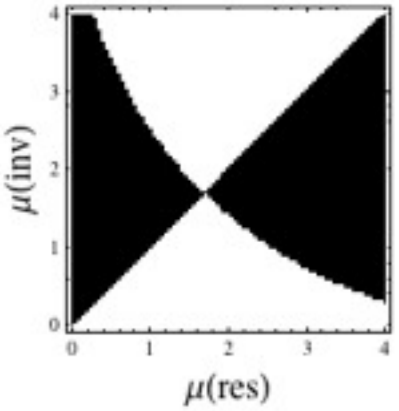

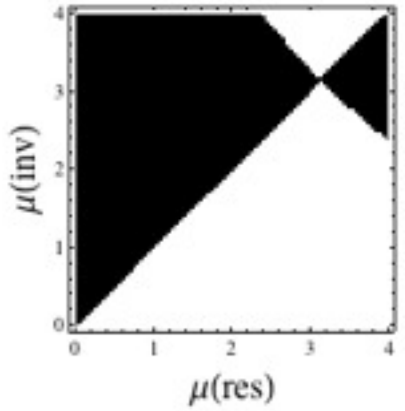

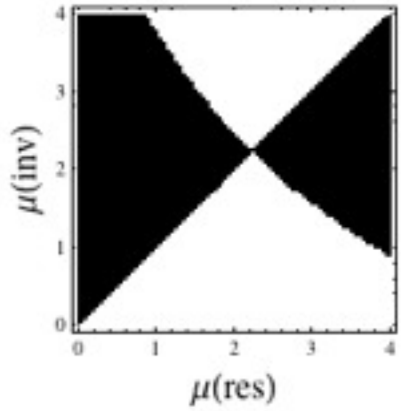

An accelerating cost also leads to an ESS with finite activation rates.

(iii) Square root cost on mortality: $m = m_0\left(1+\sqrt{\mu/\nu}\right)$. Numerical values as in (i).

$\gamma_i = 0$, $\gamma_a = 0.2$ $\gamma_i = 0$, $\gamma_a = 1$ $\gamma_i = 0.5$, $\gamma_a = 1$

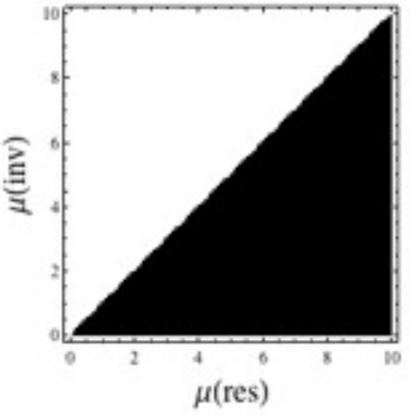

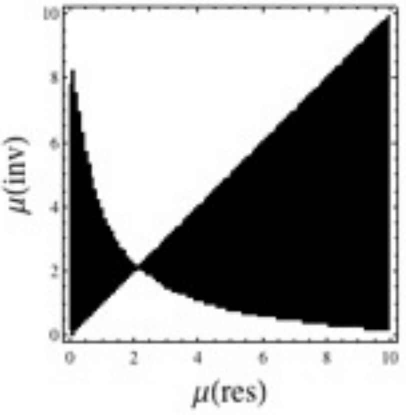

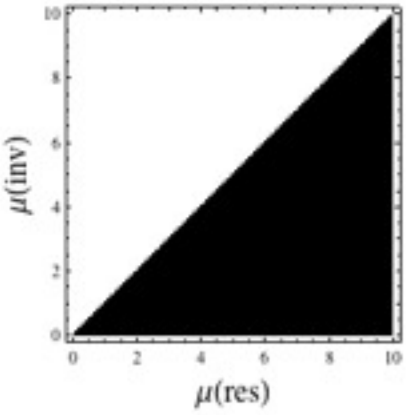

Here only a strong benefit of mounting an immune response (middle frame) gives rise to a non-zero ES activation rate. Even then, a closer look reveals that $\mu = 0$ is also an ESS (the two ESS are separated by an evolutionary repeller):

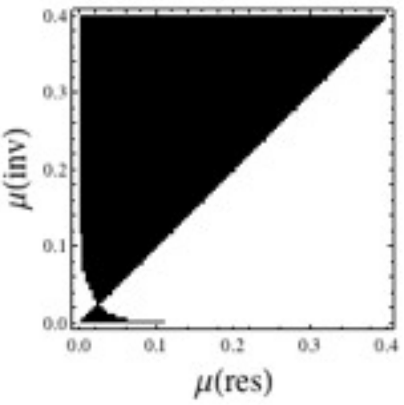

(iv) Linear cost on fecundity: $b = b_0\left(1-\mu/v\right)$. Numerical values as in Table S1 and b_0=1, v=20.

$\gamma_i = 0$, $\gamma_a = 0.2$ $\gamma_i = 0$, $\gamma_a = 1$ $\gamma_i = 0.5$, $\gamma_a = 1$

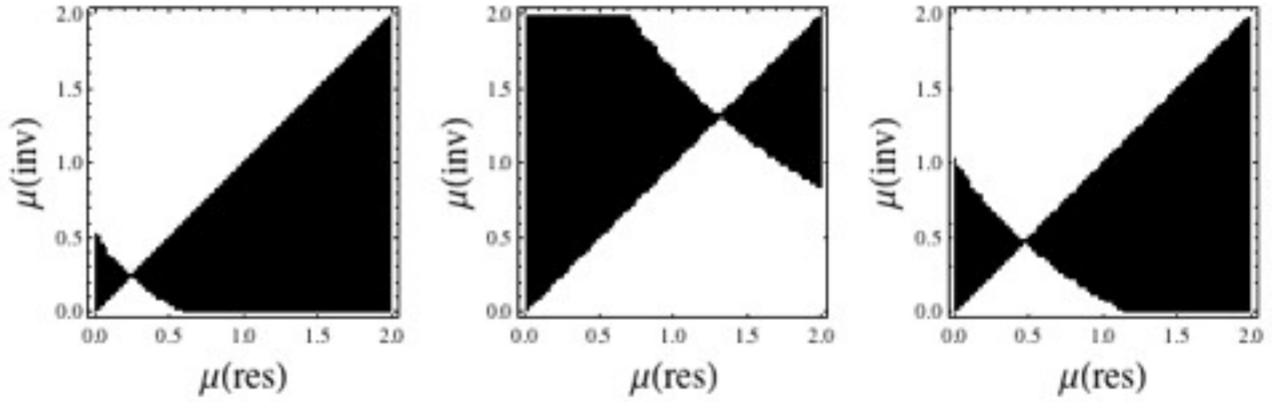

(v) Quadratic cost on fecundity: $m = b_0\left[1-\left(\mu/v\right)^2\right]$. Numerical values as in (iv).

$\gamma_i = 0$, $\gamma_a = 0.2$ $\gamma_i = 0$, $\gamma_a = 1$ $\gamma_i = 0.5$, $\gamma_a = 1$

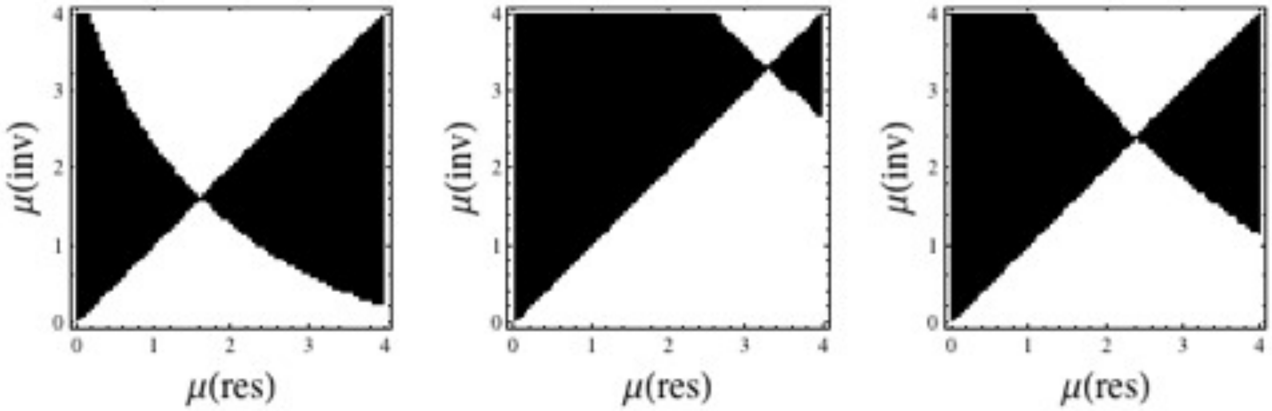

(vi) Square root cost on fecundity: $m = b_0\left[1-\sqrt{\mu/v}\right]$. Numerical values as in (iv) except v = 50.

$\gamma_i = 0$, $\gamma_a = 1$ $\gamma_i = 0$, $\gamma_a = 1.5$ $\gamma_i = 0.5$, $\gamma_a = 2$

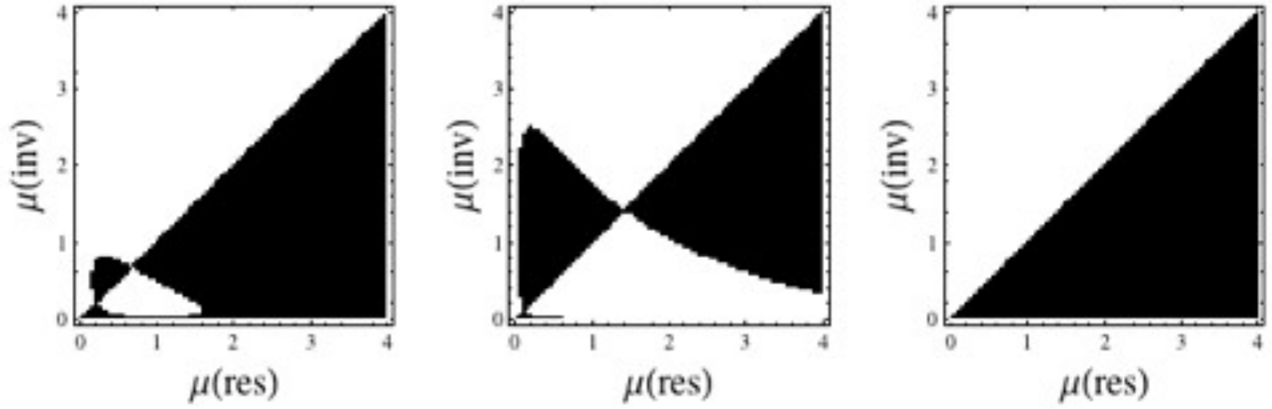

Here a non-zero ESS exists only if the cost on fecundity is very low (with a large value of v) and the benefit of the immune response very high.

b. Supplementary result

ES activation rate (μ) plotted against the two levels of pathogen virulence. Same as figure 4, except that infectivity parameters are kept constant: $\delta_i = \delta_a = 1$.

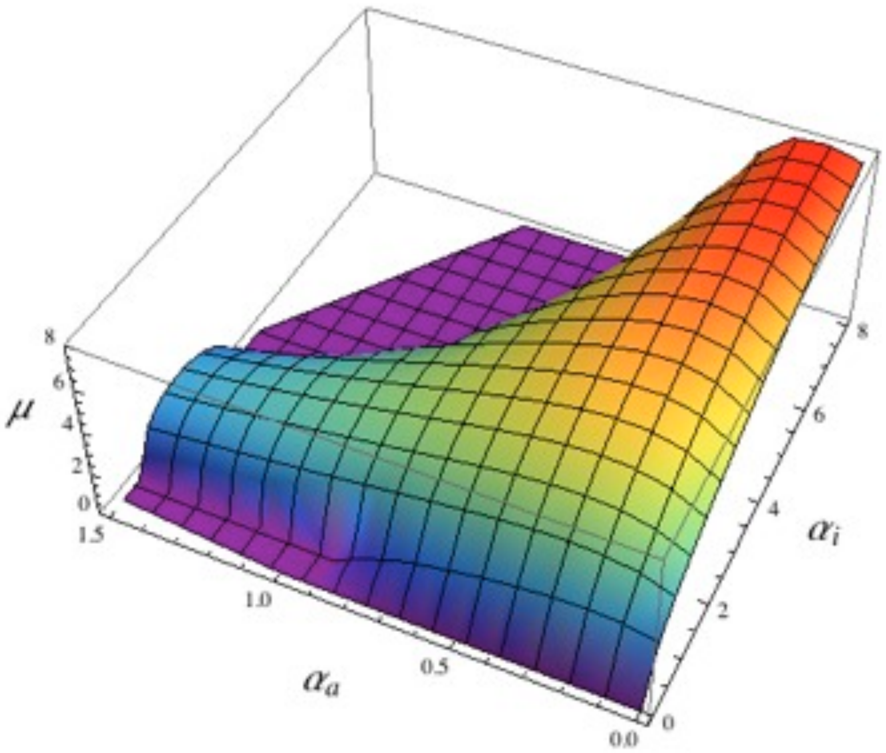

c. SIARA model

The same analyses as above can be done for the SIARA model, which is just slightly more complicated because of the extra variable. With two host genotypes we have 8 equations:

$$\begin{cases} \dfrac{dS_1}{dt} = b_1\left(S_1 + I_1 + A_1 + R_1\right)\left(1 - qN\right) - \beta S_1\left(\delta_i I_1 + \delta_a A_1 + \delta_i I_2 + \delta_a A_2\right) - m_1 S_1 + \gamma_i I_1 \\ \dfrac{dI_1}{dt} = \beta S_1\left(\delta_i I_1 + \delta_a A_1 + \delta_i I_2 + \delta_a A_2\right) - \left(m_1 + \alpha_i + \gamma_i + \mu_1\right) I_1 \\ \dfrac{dA_1}{dt} = \mu_1 I_1 + \beta R_1\left(\delta_i I_1 + \delta_a A_1 + \delta_i I_2 + \delta_a A_2\right) - \left(m_1 + \alpha_a + \gamma_a\right) A_1 \\ \dfrac{dR_1}{dt} = \gamma_a A_1 - \beta R_1\left(\delta_i I_1 + \delta_a A_1 + \delta_i I_2 + \delta_a A_2\right) - m_1 R_1 \\ \dfrac{dS_2}{dt} = b_2\left(S_2 + I_2 + A_2 + R_2\right)\left(1 - qN\right) - \beta S_2\left(\delta_i I_1 + \delta_a A_1 + \delta_i I_2 + \delta_a A_2\right) - m_2 S_2 + \gamma_i I_2 \\ \dfrac{dI_2}{dt} = \beta S_2\left(\delta_i I_1 + \delta_a A_1 + \delta_i I_2 + \delta_a A_2\right) - \left(m_2 + \alpha_i + \gamma_i + \mu_2\right) I_2 \\ \dfrac{dA_2}{dt} = \mu_2 I_2 + \beta R_2\left(\delta_i I_1 + \delta_a A_1 + \delta_i I_2 + \delta_a A_2\right) - \left(m_2 + \alpha_a + \gamma_a\right) A_2 \\ \dfrac{dR_2}{dt} = \gamma_a A_2 - \beta R_2\left(\delta_i I_1 + \delta_a A_1 + \delta_i I_2 + \delta_a A_2\right) - m_2 R_2 \end{cases}$$

As before, I assume that genotype 1 is initially on its own in the population with the pathogen and reaches its stable equilibrium $\left(S_1^*, I_1^*, A_1^*, R_1^*\right)$ before genotype 2 appears by mutation. This leads to define the hosts' reproduction matrix **F** as:

$$\mathbf{F} = \left(1 - qN_1^*\right)\begin{pmatrix} b_1 & b_1 & b_1 & b_1 & 0 & 0 & 0 & 0 \\ 0 & 0 & 0 & 0 & 0 & 0 & 0 & 0 \\ 0 & 0 & 0 & 0 & 0 & 0 & 0 & 0 \\ 0 & 0 & 0 & 0 & 0 & 0 & 0 & 0 \\ 0 & 0 & 0 & 0 & b_2 & b_2 & b_2 & b_2 \\ 0 & 0 & 0 & 0 & 0 & 0 & 0 & 0 \\ 0 & 0 & 0 & 0 & 0 & 0 & 0 & 0 \\ 0 & 0 & 0 & 0 & 0 & 0 & 0 & 0 \end{pmatrix}$$

and the transition matrix **V** as:

$$\mathbf{V} = \begin{pmatrix} m_1 + \Lambda_1^* & -\gamma_i & 0 & 0 & 0 & 0 & 0 & 0 \\ -\Lambda_1^* & m_1 + \alpha_i + \gamma_i + \mu_1 & 0 & 0 & 0 & 0 & 0 & 0 \\ 0 & -\mu_1 & m_1 + \alpha_a + \gamma_a & -\Lambda_1^* & 0 & 0 & 0 & 0 \\ 0 & 0 & -\gamma_a & m_1 + \Lambda_1^* & 0 & 0 & 0 & 0 \\ 0 & 0 & 0 & 0 & m_2 + \Lambda_1^* & -\gamma_i & 0 & 0 \\ 0 & 0 & 0 & 0 & -\Lambda_1^* & m_2 + \alpha_i + \gamma_i + \mu_2 & 0 & 0 \\ 0 & 0 & 0 & 0 & 0 & -\mu_2 & m_2 + \alpha_a + \gamma_a & -\Lambda_1^* \\ 0 & 0 & 0 & 0 & 0 & 0 & -\gamma_a & m_2 + \Lambda_1^* \end{pmatrix}$$

where $\Lambda_1^* = \beta\left[\delta_i I_1^* + \delta_a A_1^*\right]$ is the force of infection at equilibrium.

The eigenvalues of the next-generation matrix $\mathbf{FV}^{-1}$ are:

$\omega_1 = 1$

$$\omega_2 = \frac{b_2\left(1 - qN_1^*\right)\left[\left(m_2 + \alpha_i + \gamma_i + \Lambda_1^*\right)\left[\alpha_a\Lambda_1^* + m_2\left(m_2 + \alpha_a + \gamma_a + \Lambda_1^*\right)\right] + \mu_2\left(m_2 + \Lambda_1^*\right)\left(m_2 + \alpha_a + \gamma_a + \Lambda_1^*\right)\right]}{\left[\alpha_a\Lambda_1^* + m_2\left(m_2 + \alpha_a + \gamma_a + \Lambda_1^*\right)\right]\left[m_2\left(m_2 + \alpha_i + \gamma_i + \Lambda_1^*\right) + \Lambda_1^*\left(\alpha_i + \mu_2\right)\right]}$$

(S8)

As with the SIAS model, I use ω_2 as a measure of the mutant's fitness in order to produce Pairwise Invasibility Plots and calculate ESS, with a set of alternative cost functions (see next page).

(i) Linear cost on mortality: $m = m_0\left(1 + \mu / \nu\right)$. Numerical values as in Table S1 and m_0=0.1, ν=10.

$\gamma_i = 0, \gamma_a = 0.2$ $\gamma_i = 0, \gamma_a = 1$ $\gamma_i = 0.5, \gamma_a = 1$

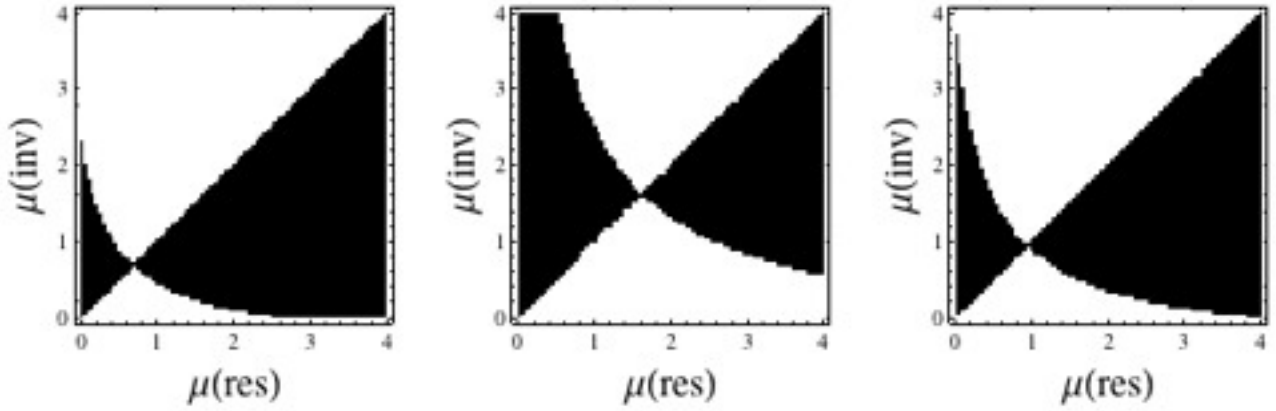

(ii) Quadratic cost on mortality: $m = m_0\left[1 + \left(\mu / \nu\right)^2\right]$. Numerical values as in (i).

$\gamma_i = 0, \gamma_a = 0.2$ $\gamma_i = 0, \gamma_a = 1$ $\gamma_i = 0.5, \gamma_a = 1$

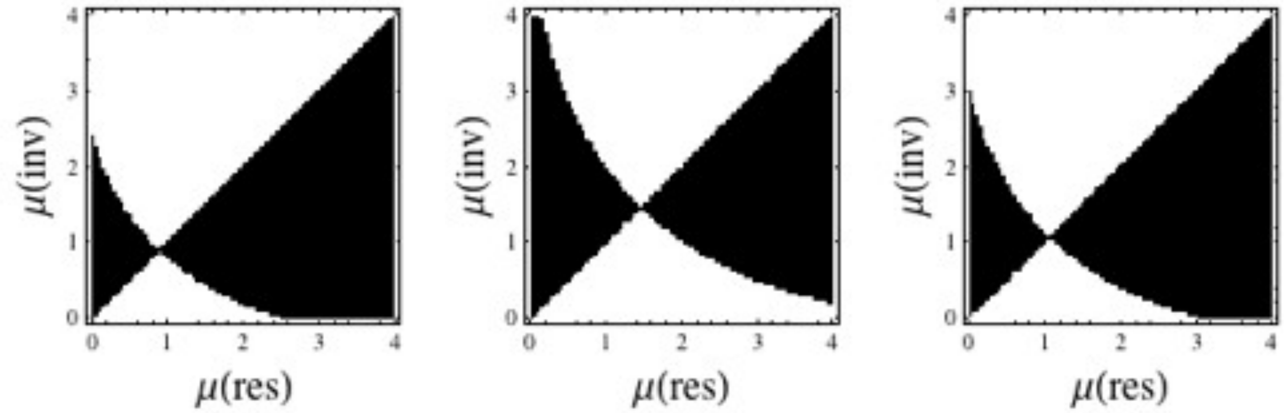

(iii) Square root cost on mortality: $m = m_0\left(1 + \sqrt{\mu / \nu}\right)$. Numerical values as in (i).

$\gamma_i = 0, \gamma_a = 0.2$ $\gamma_i = 0, \gamma_a = 1$ $\gamma_i = 0.5, \gamma_a = 1$

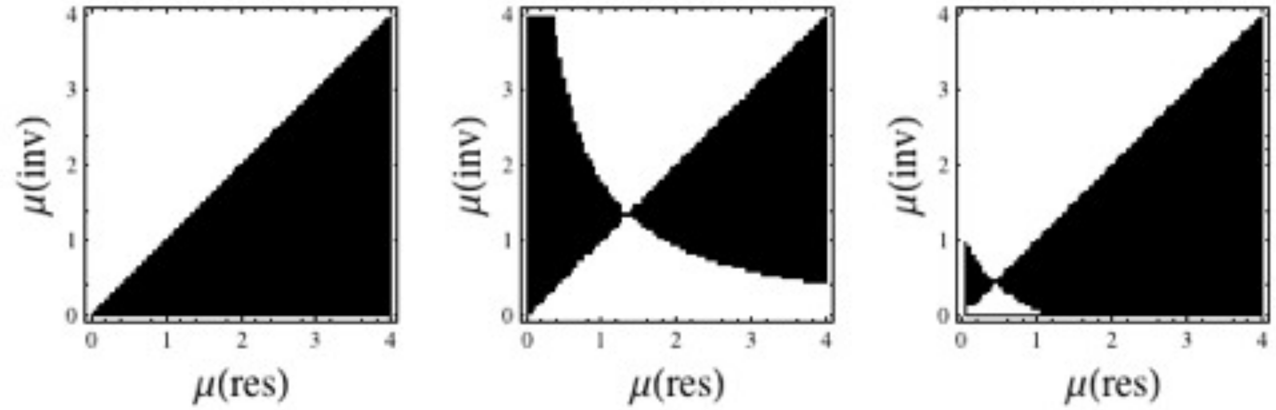

(iv) Linear cost on fecundity: $b = b_0\left(1 - \mu / v\right)$. Numerical values as in Table S1 and b_0=1, v=20.

$\gamma_i = 0, \gamma_a = 0.2$ $\gamma_i = 0, \gamma_a = 1$ $\gamma_i = 0.5, \gamma_a = 1$

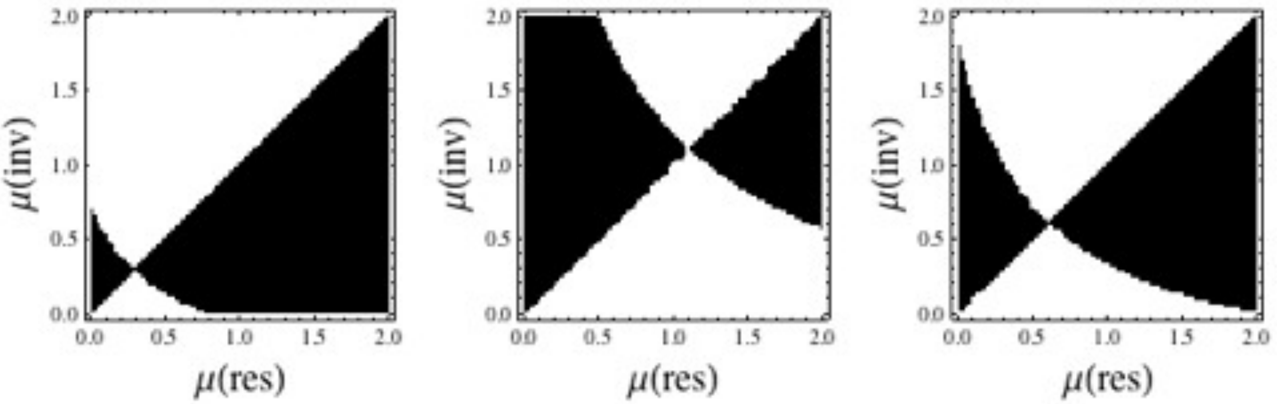

(v) Quadratic cost on fecundity: $b = b_0\left[1 - \left(\mu / v\right)^2\right]$. Numerical values as in (iv).

$\gamma_i = 0, \gamma_a = 0.2$ $\gamma_i = 0, \gamma_a = 1$ $\gamma_i = 0.5, \gamma_a = 1$

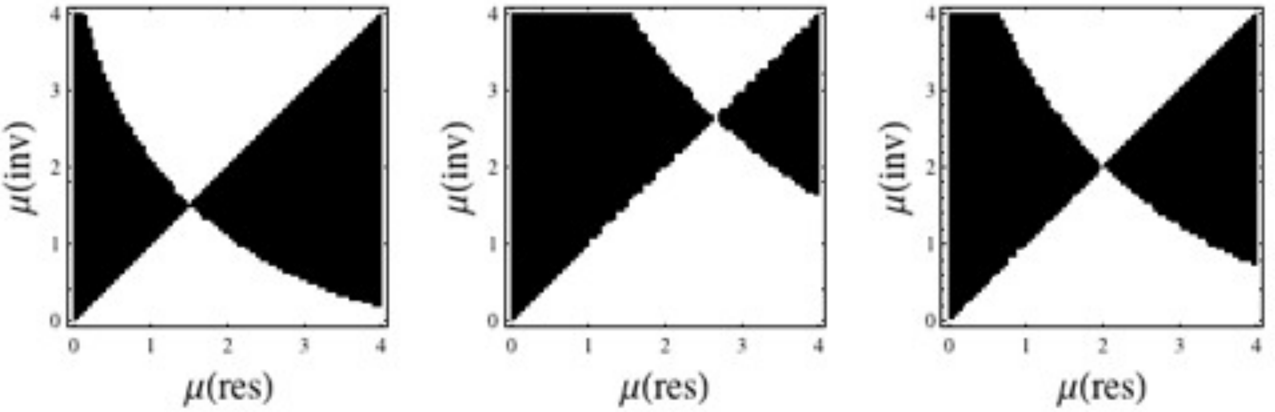

(vi) Square root cost on fecundity: $b = b_0\left[1 - \sqrt{\mu / v}\right]$. Numerical values as in (iv) except v = 50.

$\gamma_i = 0, \gamma_a = 1$ $\gamma_i = 0, \gamma_a = 1.5$ $\gamma_i = 0.5, \gamma_a = 2$

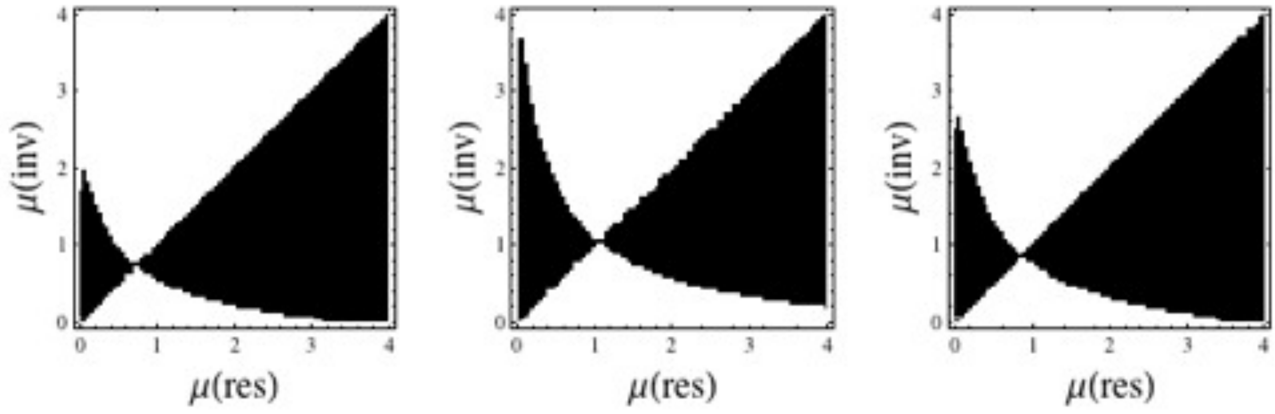

5. Coevolution

As explained in the main text, I have chosen to model coevolution by modifying the host evolutionary algorithm under the assumption that the pathogen's phenotype is at the ESS with respect to the resident host's phenotype. The fitness of a mutant host genotype is still given by expression (S7) or (S8) above (respectively for the SIAS and SIARA models), but parameters δ_i, δ_a, α_i and α_a are now functions of the resident host's genotype and other parameters as determined in Section 3. Under this scenario, any ESS for the host will automatically be associated with a corresponding ESS for the pathogen, resulting in a Co-Evolutionary Stable Strategy (CoESS).

Here I will give a few examples of PIPs for the host, as I did in section 4, but also considering the two models of pathogen evolution: plastic virulence or fixed virulence.

a. SIAS model, plastic virulence

(i) <u>Linear cost on mortality</u>: $m = m_0(1 + \mu / v)$. Numerical values as in Table S1 and m_0=0.1, v=10, δ_0=1, ε=1.

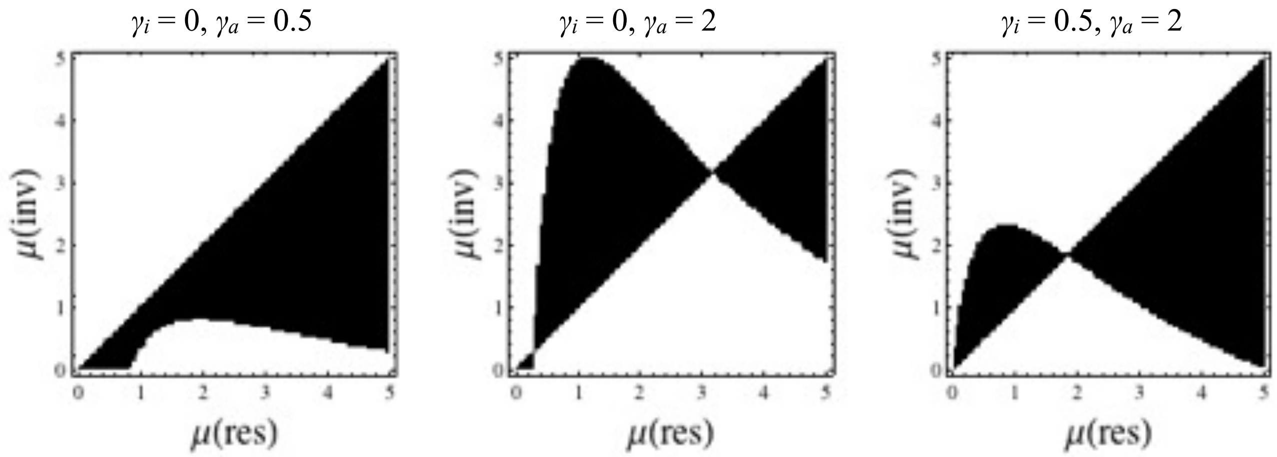

The central panel clearly shows the two ESS ($\mu \approx 3$ and $\mu = 0$) separated by a repeller ($\mu \approx 0.3$), as explained in the main text (section 3c) and illustrated in a different way on Fig. 4A. As we increase the value of γ_i the repeller drops to 0 (right-hand side panel) so the lower ESS, while it still exists, is no longer stable by convergence (a so-called 'Garden of Eden' singularity [5]).

(ii) <u>Linear cost on fecundity</u>: $b = b_0(1 - \mu / v)$. Numerical values as in Table S1 and b_0=1, v=20.

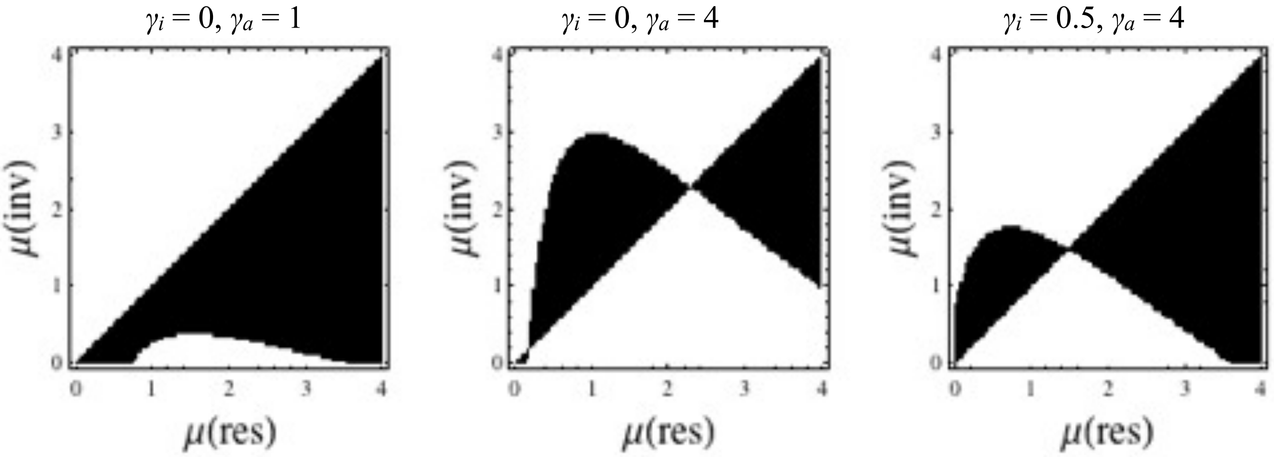

b. SIAS model, fixed virulence

(i) Linear cost on mortality: $m = m_0(1+\mu/v)$. Numerical values as in Table S1 and m_0=0.1, v=10, δ_0=1, ε=1.

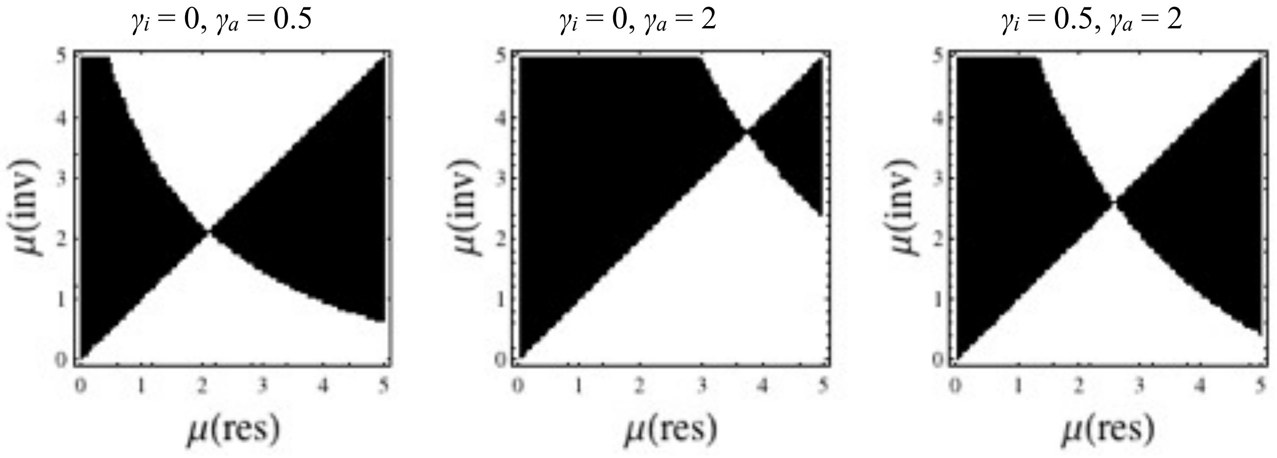

(ii) Linear cost on fecundity: $b = b_0(1-\mu/v)$. Numerical values as in Table S1 and b_0=1, v=20.

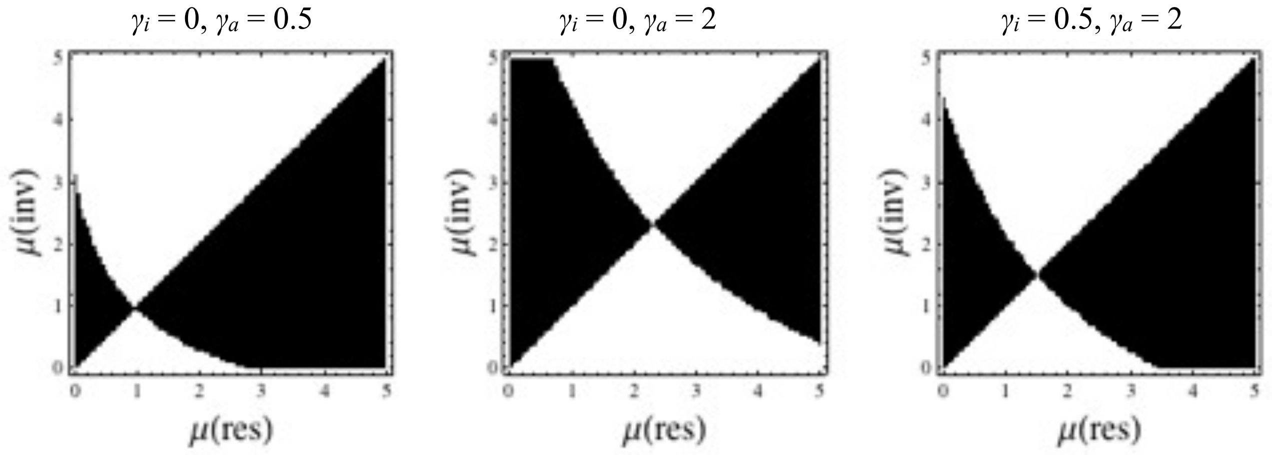

c. SIARA model, plastic virulence

(i) Linear cost on mortality: $m = m_0(1+\mu/v)$. Numerical values as in Table S1 and m_0=0.1, v=10, δ_0=1, ε=1.

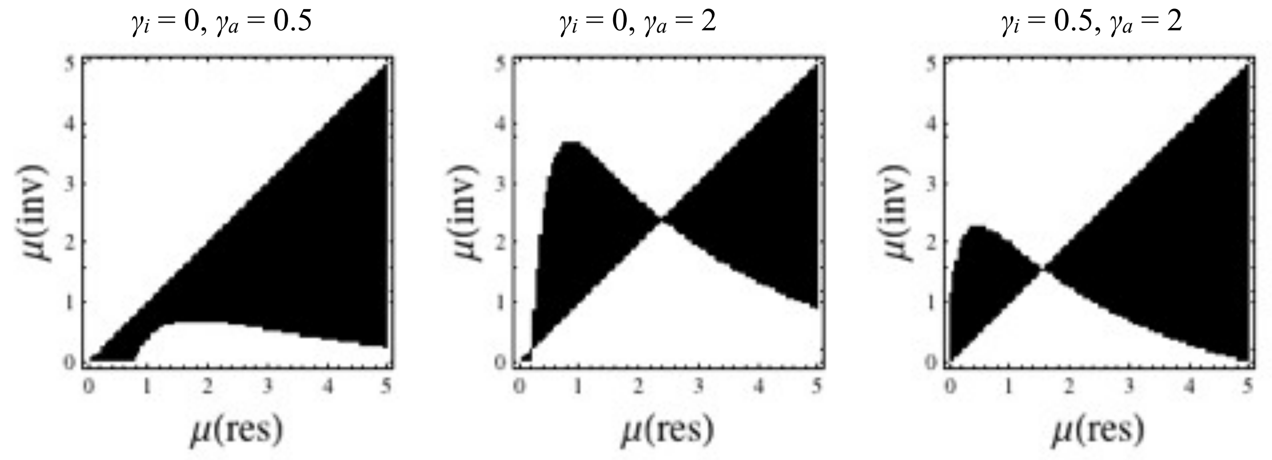

(ii) <u>Linear cost on fecundity</u>: $b = b_0(1 - \mu / v)$. Numerical values as in Table S1 and b_0=1, v=20.

$\gamma_i = 0, \gamma_a = 1$ $\gamma_i = 0, \gamma_a = 4$ $\gamma_i = 0.5, \gamma_a = 4$

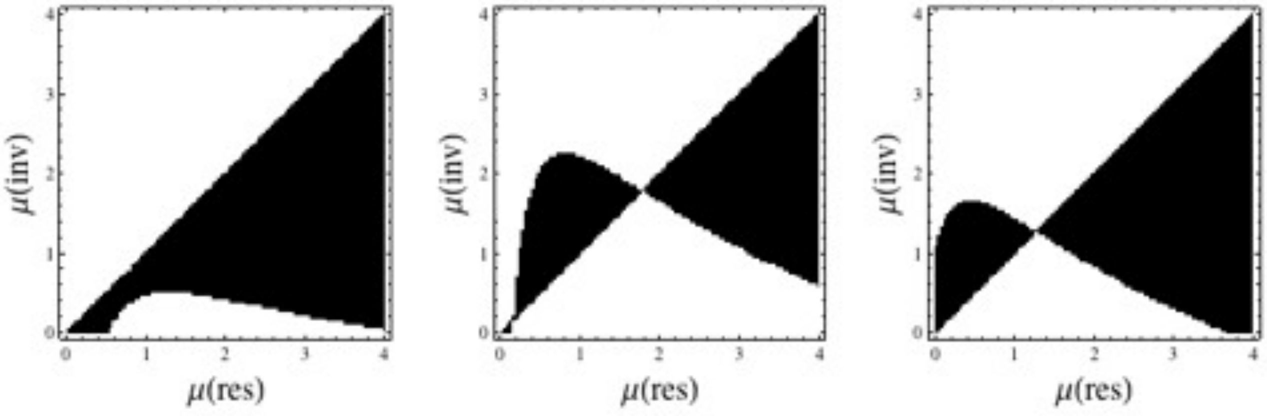

d. SIARA model, fixed virulence

(i) <u>Linear cost on mortality</u>: $m = m_0(1 + \mu / v)$. Numerical values as in Table S1 and m_0=0.1, v=10, δ_0=1, ε=1.

$\gamma_i = 0, \gamma_a = 0.5$ $\gamma_i = 0, \gamma_a = 2$ $\gamma_i = 0.5, \gamma_a = 2$

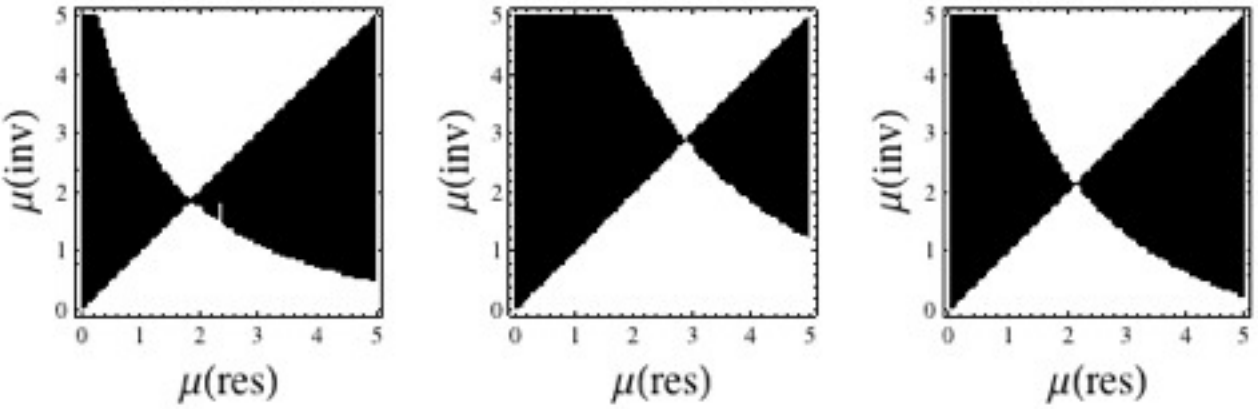

(ii) <u>Linear cost on fecundity</u>: $b = b_0(1 - \mu / v)$. Numerical values as in Table S1 and b_0=1, v=20.

$\gamma_i = 0, \gamma_a = 0.5$ $\gamma_i = 0, \gamma_a = 2$ $\gamma_i = 0.5, \gamma_a = 2$

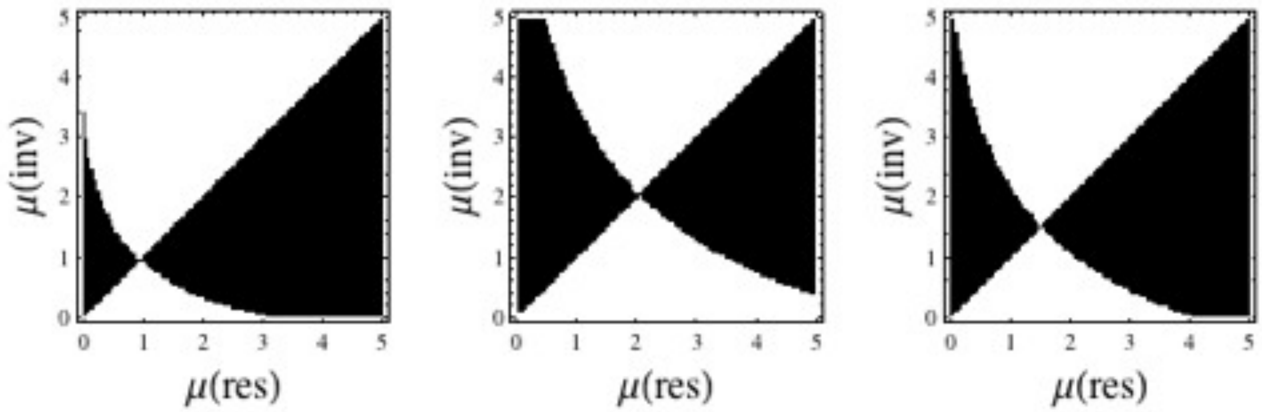

Bibliography

1 Hurford, A., Cowden, D. & Day, T. 2010 Next-generation tools for evolutionary invasion analyses. *J. R. Soc. Interface* **7**, 561-571.

2 Bremermann, H. J. & Pickering, J. 1983 A game-theoretical model of parasite virulence. *J. Theor. Biol.* **100**, 411-426.

3 van Baalen, M. & Sabelis, M. W. 1995 The scope for virulence management: a comment on Ewald's view on the evolution of virulence. *Trends Microbiol.* **3**, 414-416.

4 Geritz, S. A. H., Kisdi, E., Mesze, G. & Metz, J. A. J. 1997 Evolutionarily singular strategies and the adaptive growth and branching of the evolutionary tree. *Evol. Ecol.* **12**, 35-57.

5 de Mazancourt, C. & Dieckmann, U. 2004 Trade-Off Geometries and Frequency-Dependent Selection. *Am. Nat.* **164**, 765-778.
